# Mapping research hotspots and trends in gut microbiota and autoimmune diseases since the 21st century: a bibliometric visualization analysis based on two databases

**DOI:** 10.3389/fimmu.2026.1793126

**Published:** 2026-09-18

**Authors:** Pengrui Feng, Xiao Fu, Chaoyi Zhang, Dong Wang, Zan Chen, Guanglin Wang

**Affiliations:** 1 Department of Orthopaedics, West China Hospital, West China School of Medicine, Sichuan University, Chengdu, Sichuan, China; 2 West China Second University Hospital, Sichuan University, Chengdu, Sichuan, China; 3 Key Laboratory of Birth Defects and Related Diseases of Women and Children, Ministry of Education, Sichuan University, Chengdu, Sichuan, China; 4 Trauma Medical Center, Department of Orthopedics Surgery, West China Hospital, Sichuan University, Chengdu, Sichuan, China

**Keywords:** autoimmune diseases, bibliometrics, Citespace, gut microbiota, immune response, publications, VOSviewer

## Abstract

**Background:**

The gut microbiota is recognized as essential for maintaining immune homeostasis, and dysbiosis of the gut microbiota has been increasingly associated with the pathogenesis of various autoimmune diseases. Emerging evidence suggests that alterations in gut microbial composition may contribute to dysregulation of the immune system, thereby promoting inflammatory responses and autoimmunity. Despite growing interest in this field, a comprehensive bibliometric analysis integrating global research trends, influential publications, and key contributors, as well as clinical translation perspectives, remains lacking. This study aims to systematically evaluate the research landscape on the relationship between the gut microbiota and autoimmune diseases, thereby providing insights into research development, emerging hotspots, and future directions.

**Methods:**

Global publications on gut microbiota and autoimmune diseases were systematically retrieved and extracted from the Web of Science Core Collection (WOSCC) database, covering the period from January 2000 to February 2025. The literature search and data extraction were conducted in February 2025, which was predefined as the cutoff date for data collection, thereby ensuring dataset reproducibility. Publications from 2025 were treated as partial-year data due to incomplete annual coverage and were excluded from longitudinal trend analyses to minimize temporal bias. In addition, a separate search of the PubMed database was conducted to identify clinical trials in this field. Bibliometric analyses and visualization were performed using VOSviewer, CiteSpace, and R version 4.4.0.

**Results:**

A total of 3,004 publications were retrieved from the Web of Science Core Collection and 85 clinical trials from PubMed, covering the period from 2000 to 2025. The overall number of publications has steadily increased over time. Citation analysis indicates that the United States and China are the leading countries in terms of publication output and citation frequency. Harvard Medical School was identified as the most productive institution, while New York University received the highest citation counts. Ochoa-Reparaz, J was the most prolific author, whereas citation frequency was highest for Atarashi, K, followed by Ochoa-Reparaz, J. Frontiers in Immunology published the highest number of articles in this field. Keyword and literature analyses identified mechanistic links between gut dysbiosis and autoimmune diseases, clinical relevance, and causal inference as major research themes. Integration of clinical trial evidence revealed that these basic research themes have progressively translated into disease-specific investigations, particularly in type 1 diabetes mellitus, multiple sclerosis, and rheumatoid arthritis. Probiotic supplementation, dietary modulation, and fecal microbiota transplantation represented emerging microbiota-targeted strategies, highlighting the transition of this field from mechanistic exploration toward clinical intervention.

**Conclusion:**

While previous bibliometric studies have primarily relied on a single database, the present study integrates bibliometric data from the Web of Science Core Collection with supplementary clinical trial data retrieved from PubMed. This study provides valuable reference for identifying research foci and highlighting future research directions.

## Introduction

Autoimmune diseases comprise a heterogeneous group of chronic disorders characterized by aberrant immune responses against self-antigens, leading to tissue damage, systemic inflammation, and significant global morbidity (1). The global prevalence of autoimmune diseases has increased over recent decades, reflecting complex interactions between genetic susceptibility, environmental exposures, and immune dysregulation (2).

In this context, the human gut microbiota, which is a dense, dynamic community of bacteria, archaea, viruses, and fungi residing in the gastrointestinal tract, has been recognized as an essential mediator of host immune homeostasis and systemic health (3). The gut microbiota has been shown to influence immune system development, intestinal barrier integrity, and metabolic signaling, thereby modulating host responses to exogenous and endogenous antigens (3, 4). Perturbations of this microbial ecosystem have been consistently associated with a spectrum of autoimmune diseases, including rheumatoid arthritis (5), systemic lupus erythematosus (6), type 1 diabetes (7), multiple sclerosis (8), and inflammatory bowel diseases (9, 10). Mechanistic studies suggest that dysbiosis may promote autoimmune processes through increased intestinal permeability, altered microbial metabolite profiles, molecular mimicry, and pro-inflammatory immune modulation (10).

The rapid growth of research at the interface of gut microbiota and autoimmune diseases has highlighted the need for bibliometric evaluation to characterize developmental trajectories, knowledge structures, and emerging research themes. Bibliometric analysis—a set of mathematical and statistical methods used to assess publication patterns, scientific productivity, and thematic evolution—has been widely applied to map complex research landscapes across disciplines (11). Through analyses of co-authorship networks, keyword co-occurrence, citation dynamics, and geographical distribution, bibliometric approaches enable the identification of influential contributions, latent trends, and thematic shifts over time. Such analytical frameworks have been widely applied and have provided valuable insights in fields ranging from microbiome research to immunology and chronic diseases (12, 13).

Despite the substantial body of primary research linking gut microbial profiles with autoimmune conditions, comprehensive bibliometric assessments in this domain remain limited. A recent bibliometric and visual analysis of studies published between 2004 and 2022 reported substantial growth in publications, with major contributions from North American and European institutions, and identified core themes including dysbiosis, short-chain fatty acids, probiotics, and immune cell interactions (14). However, substantial knowledge gaps persist with respect to evolving research hotspots, the translation of basic research into clinical practice, cross-disease microbial signatures, interdisciplinary collaborations, and future research frontiers.

To address this gap, the present study presents an updated bibliometric analysis of research on gut microbiota and autoimmune diseases with an expanded methodological scope. Compared with previous bibliometric studies, particularly Zhang et al. (2023), which focused solely on Web of Science–indexed publications from 2004 to 2022, this study extends the analytical framework in three key aspects. First, the dataset was updated to include more recent publications, providing a more up-to-date overview of research developments in this rapidly evolving field. Second, in addition to the Web of Science Core Collection, PubMed-derived clinical trial data were incorporated, enabling a dual-database perspective capturing both scientific production and clinical translation. Third, a translational mapping framework was introduced to integrate bibliometric hotspots with clinical trial evidence, allowing evaluation of gaps between basic research and clinical application. Through this multi-layered approach, the present study characterizes research trends and provides insights into the translational trajectory of gut microbiota research in autoimmune diseases.

## Methods

### Database and search strategy

The bibliographic data were retrieved from the Web of Science Core Collection (WoSCC), covering publications indexed from January 1, 2000 to February 1, 2025. To avoid bias caused by continuous database updates, all searches were completed on a single day (March 12, 2025). The initial search identified 3,181 records. Eligibility screening was performed, limiting inclusion to original articles and reviews published in English. Publication types such as book chapters, conference abstracts, reprints, and news items were excluded. After applying these criteria, 3,004 records were included for further analysis. The study selection workflow is illustrated in **Figure 1**. A comprehensive search strategy was constructed using Boolean operators, and the initial retrieval was further refined through manual screening of titles and abstracts to reduce irrelevant records (**Supplementary Table 1)**. PubMed was subsequently searched using a predefined strategy, with inclusion limited to clinical trials only (**Supplementary Table 1)**. All data extraction procedures were independently conducted by two investigators to ensure consistency, with discrepancies resolved through discussion.

**Figure 1:**
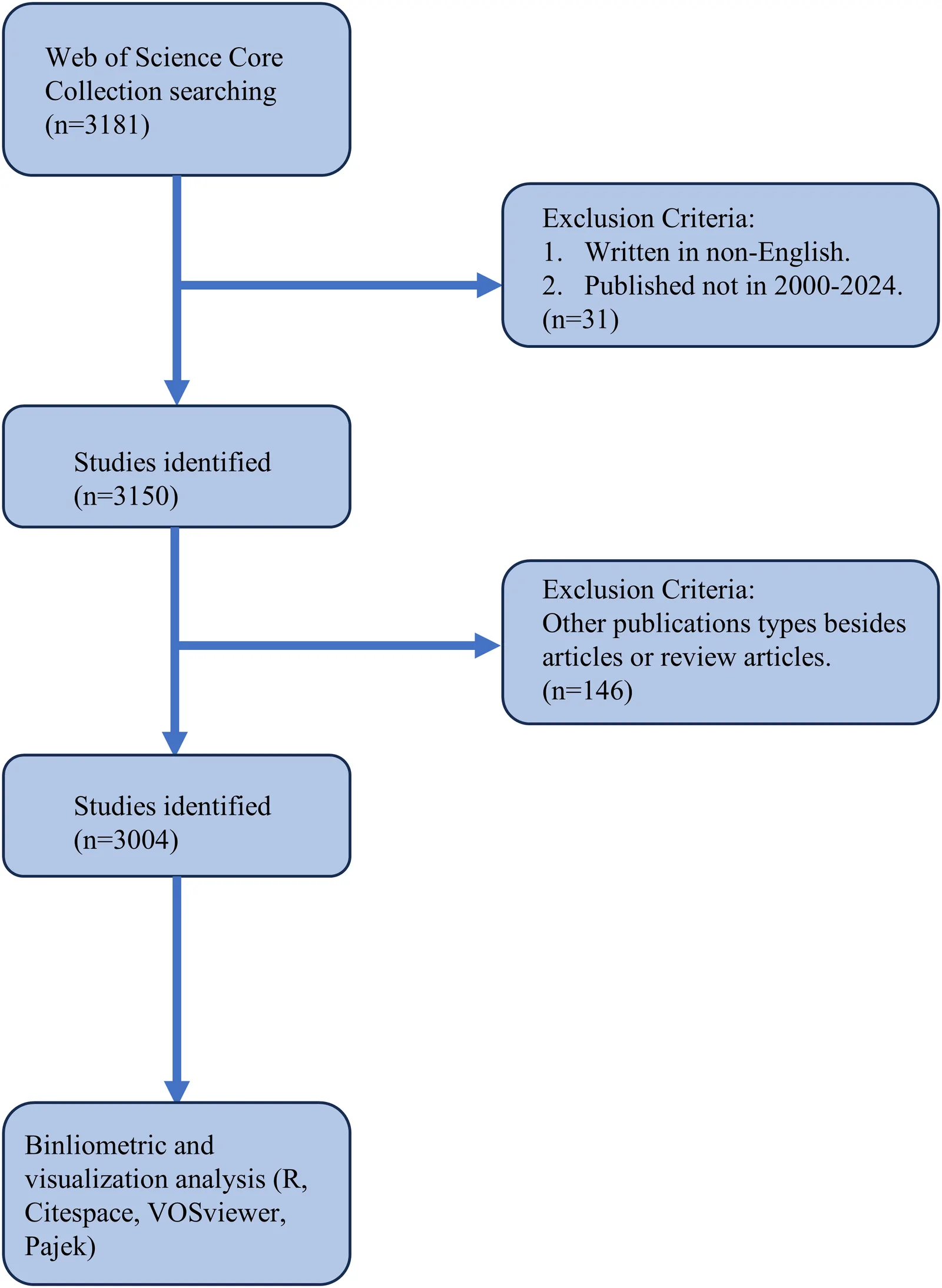
PRISMA flow diagram illustrating the study selection process for the bibliometric analysis of gut microbiota and autoimmune diseases. The diagram presents the identification, screening, eligibility assessment, and inclusion procedures of publications retrieved from the Web of Science Core Collection and clinical trials identified from PubMed.

### Data analysis

The dataset retrieved from the Web of Science Core Collection (WoSCC) was analyzed using VOSviewer (v1.6.20), CiteSpace (v6.4.R1), and the Bibliometrix R package. These tools were selected due to their complementary functionalities in bibliometric analysis. VOSviewer was used to construct and visualize bibliometric networks, enabling mapping of citation and co-citation relationships. CiteSpace was used for co-citation analysis and identification of temporal patterns and emerging research trends. In parallel, the Bibliometrix R package facilitated data cleaning, structuring, and quantitative analysis of bibliographic records. The integration of these tools enabled multidimensional visualization and interpretation of the dataset, an approach widely used in bibliometric studies. Given the bias introduced by incomplete-year data, 2025 publications were treated as a right-censored time point in the longitudinal analysis. To ensure robust temporal trend estimation, only complete calendar years (2000–2024) were included in regression-based trend analysis, while 2025 data were retained for descriptive analysis.

## Results

### Annual publications and citations trends

**Figure 2A** illustrates the temporal trends in annual publications and citation frequency from 2000 to 2025. Overall, the number of annual publications increased progressively over the study period, although slight declines were observed in 2003, 2007, and 2022. The highest annual publication output was recorded in 2024, with 406 articles published. The annual citation frequency also demonstrated an upward trajectory, with the most pronounced increase observed between 2020 and 2021. Publications published in 2022 exhibited the highest average annual citation frequency. Clinical trials retrieved from PubMed demonstrated a similar temporal pattern. **Figure 2B** presents the annual publication trends of research articles from 2000 to 2024 together with the fitted logistic regression curve. The number of publications increased continuously throughout the study period. A noticeable inflection point was identified between 2015 and 2018, followed by a marked acceleration in publication growth that peaked in the early 2020s.

**Figure 2:**
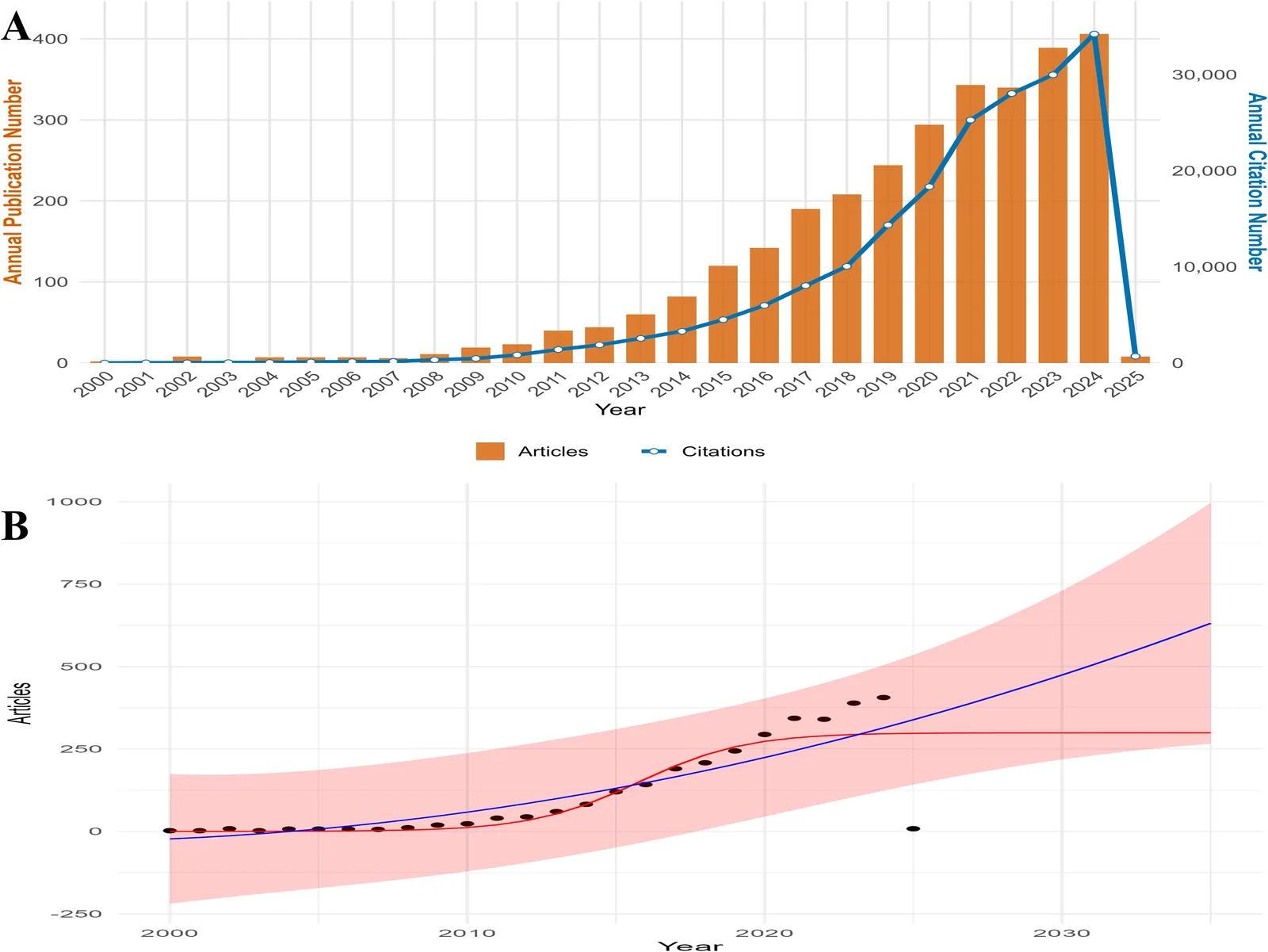
Description of publication volume and citation frequency and forecast of publication volume. **(A)** The number of publications and citation frequency for each year from 2000 to 2025, with the overall increasing trend of gut microbiota and autoimmune diseases publications and citation frequency reaching the maximum in 2024 (2025 data only until February 27). **(B)** Illustrates the annual publication trend of research articles in this field from 2000 to 2024, along with a logistic regression fitting curve. The black dots represent the actual annual publication counts, while the blue curve denotes the fitted logistic growth model, and the red line indicates the prediction plateau with a 95% confidence interval (shaded area).

### Geographical distribution and collaborative patterns of research

A total of 98 countries/regions contributed publications to research on gut microbiota and autoimmune diseases. **Figure 3A** illustrates that international collaboration links were mainly concentrated among North America, Europe, East Asia, and Oceania, with the strongest collaboration observed between North America and Europe. Countries with close collaboration relationships were clustered according to the same colors in **Figure 3B**. **Table 1** shows the top 10 countries/regions ranked by publication output, citation frequency, and betweenness centrality. To evaluate the influence of different countries within the collaboration network, betweenness centrality was calculated using CiteSpace. This metric reflects the role of individual countries as bridges connecting different research communities. Higher betweenness centrality values indicate a more prominent bridging role within the collaboration network. The United States ranked first in publication output (1009 articles), followed by China (661 articles) and Italy (313 articles). The United States also had the highest citation frequency (86,964), substantially exceeding those of Germany and Italy. Regarding betweenness centrality, the United States ranked first within the network, with a value of 0.28.

**Figure 3:**
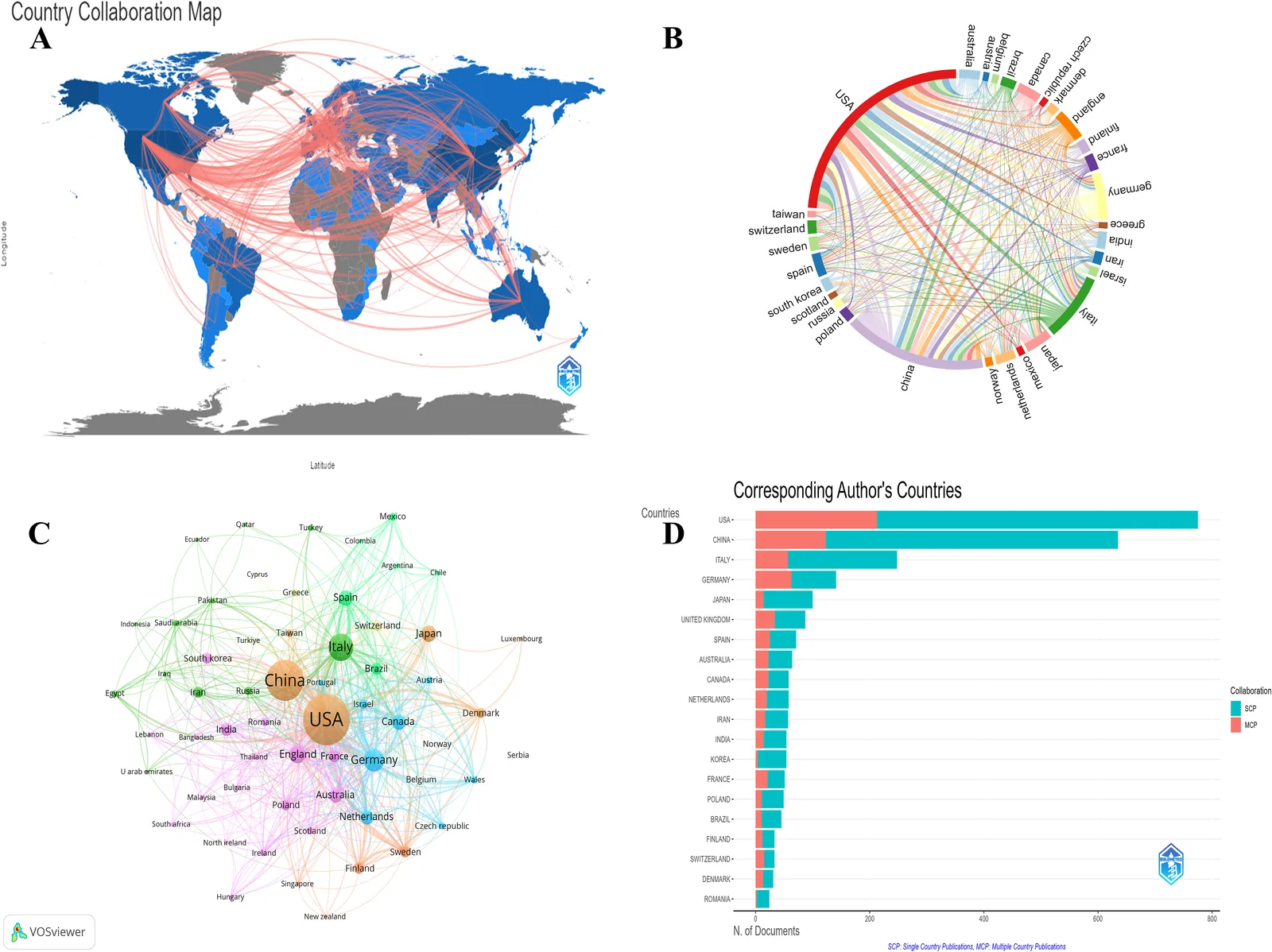
Analysis of gut microbiota and autoimmune diseases-related country/region. **(A)** Countries/regions involved in gut microbiota and autoimmune diseases-related research. The links between countries/regions indicate their collaborations and connections. The greater the number of links, the closer the collaboration between the countries/regions. **(B)** Visualized cooperative relations between countries/regions. The thickness of the lines connecting these countries is indicative of the strength of their collaborative relationships; thicker lines denote stronger ties, while thinner lines suggest less frequent collaboration. **(C)** Analysis of collaborative network visualization of countries/regions in VOSviewer. **(D)** Illustrates the distribution of countries and the collaborative efforts among corresponding authors.

**Table 1:** The top 10 countries/regions in terms of the number of articles published.

Rank	Countries/regions	Publications	Citations	Centrality
1	USA	1009	86964	0.28
2	China	661	18002	0.10
3	Italy	313	15185	0.12
4	Germany	219	17914	0.07
5	England	146	13509	0.09
6	Japan	125	12777	0.02
7	Canada	111	9575	0.03
8	Spain	109	7997	0.02
9	Australia	98	7646	0.05
10	Netherlands	95	5138	0.01

**Figure 3C** presents the global collaboration network generated using VOSviewer. Countries and regions were classified into eight clusters according to collaboration strength, with each cluster represented by a distinct color. **Figure 3D** illustrates the ranking of the top 20 countries based on corresponding author publication output. China and the United States ranked first and second in publication output, respectively. China showed a greater proportion of single-country publications (SCP), whereas the United States exhibited a higher proportion of multiple-country publications (MCP), indicating stronger international collaboration. Other countries, including Germany, Italy, the United Kingdom, Spain, and Australia, also demonstrated active participation in international collaborative research.

### Analysis of institutional distribution and collaborative networks

**Table 2**; **Supplementary Table 2** exhibit the top ten organizations in terms of number of publications and citation frequency using VOSviewer and Citespace statistics. The institution with the most publications is Harvard University (76), which also has a high centrality value of 0.08. It means Harvard University generate a significant influence in the academic collaboration network. And this is closely followed by Yale University (39) and Shanghai Jiao Tong University (38), both of which have a centrality value of 0.03. Notably, six of the top ten organizations in terms of number of publications belong to the United States, accounting for more than half. About citation frequency, the most frequently cited institution is New York University (11507), followed by Harvard University (9656) and University of California San Francisco (8731). The top 10 most cited institutions are from the United States with. Furthermore, the centrality of Harvard University is 0.11, which demonstrates this institution occupies a significant position in research in the field of gut microbiota and autoimmune diseases.

**Table 2:** Top 10 institutions in terms of number of articles issued and the corresponding centrality.

Rank	Countries/regions	Publications	Centrality
1	Harvard Medical School	76	0.08
2	Yale University	39	0.03
3	Shanghai Jiao Tong University	38	0.03
4	New York University	37	0.03
5	Mayo Clinic	35	0.02
6	Harvard University	34	0.11
7	University of Florida	34	0.01
8	University of Milan	34	0.03
9	University of helsinki	32	0.05
10	University of Toronto	31	0.03

In VOSviewer, the network of cooperation between institutions was divided into 8 clusters with different colors (**Figure 4A**). **Figure 4B** is the overlay visualization of institutional distribution and collaborative network. We can conclude that the numbers of publications conducted by Chinese Academy of Sciences, Central South University, Peking University and other organizations have significant increase in recent years. In contrast, the nodes represented by Harvard University, University of Chicago and some other institutions have a blue color bias, indicating that these institutions have been inactive recently. Overall, Chinese organizations are mostly active in recent years, while European and American organizations have a much longer time horizon. **Supplementary Figure 1** is result of analysis of collaborative network visualization of institutions in CiteSpace. We can observe that Harvard university has the most publications, but its centrality is low. On the contrary, University of California System and National institutes of Health have large centrality value.

**Figure 4:**
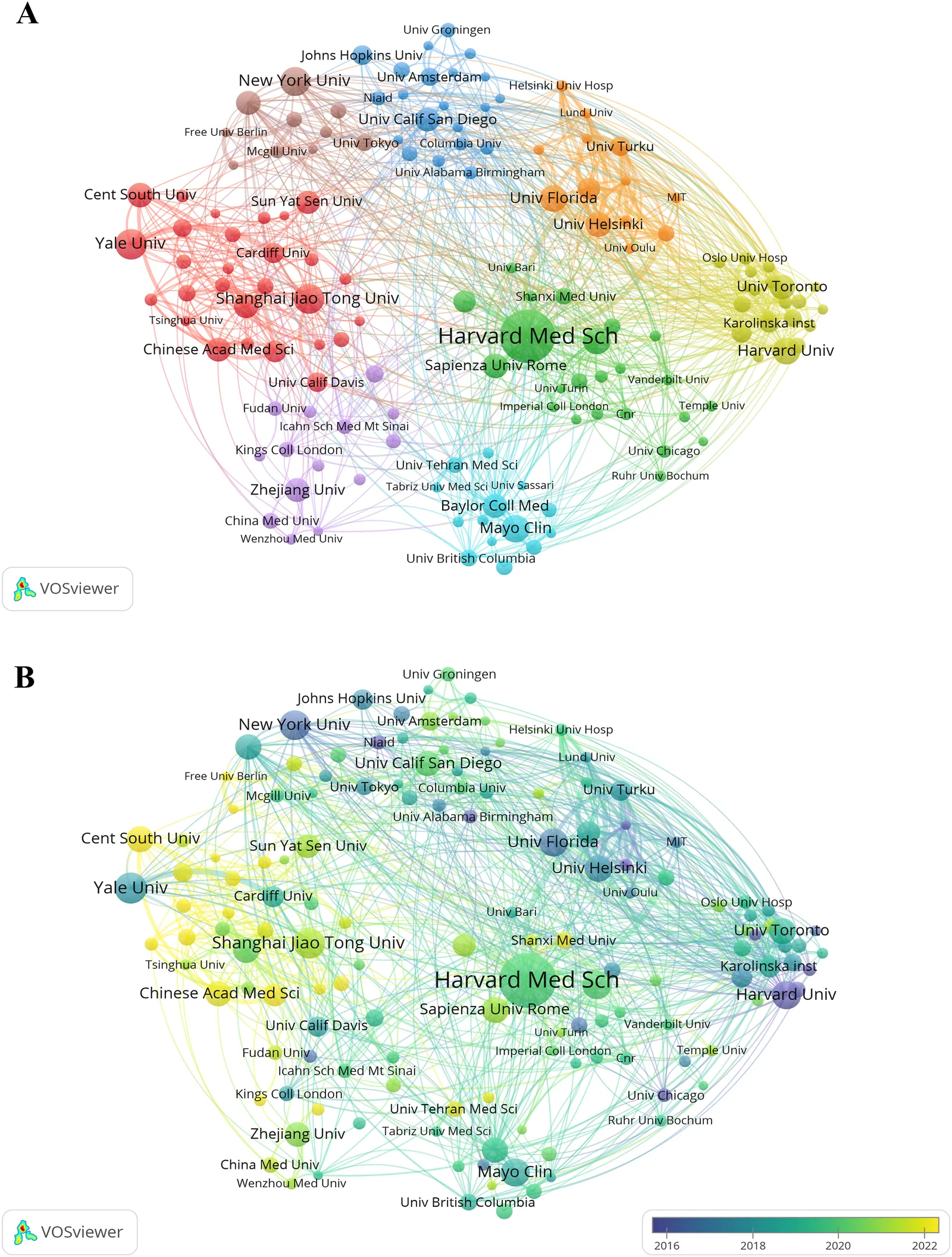
Analysis of gut microbiota and autoimmune diseases-related institution. **(A)** Analysis of collaborative network visualization of institutions in VOSviewer. **(B)** Analysis of the number of articles published by institutions in recent years. The recent 5 years heat value of each institution is obtained by dividing the number of publications in recent 5 year by the total number of publications. The size of the node represents the amount of documents published by the organization, the larger the node means more documents are published. The color of the node symbolizes the average publication time (the more yellowish the color, the newer the study; the more blue, the earlier the study).

### Analysis of authors’ distribution and citation collaborative networks

Total link strength (TLS) refers to the total strength of the co-authorship links of one author with others in the network. The higher the TLS value, the stronger the collaborative relationships (15). Total link strength (TLS) refers to the total strength of the co-authorship links of one author with others in the network. The higher the TLS value, the stronger the collaborative relationships 16. **Table 3** shows the highest number of publications is by Ochoa-Reparaz, Javier, located at Boise State University, while his high TLS (708) values reflect the strong collaborative relationships. Close behind in terms of publications are Wen, Li (18, Yale University) and Kasper, Lloyd H (17, University of Michigan). **Supplementary Table 3** demonstrates that the author with the highest co-citation value is Atarashi, K (655, Keio University School of Medicine), followed by Ochoa-reparaz, J (630, Boise State University) and Berer, K, (587, Max Planck Institute of Neurobiology). The authors from USA make up the largest share of the top 10 authors in terms of publications and frequency of co-citations. Co-citation analysis further revealed distinct author clusters, suggesting the presence of multiple interconnected research communities (**Figure 5**).

**Table 3:** Top 10 authors in terms of number of publications, the corresponding institutions and total link strength.

Rank	Authors	Publications	Institutions	Total link strength
1	Ochoa-Reparaz, Javier	19	Boise State University (USA)	708
2	Wen, Li	18	Yale University (USA)	446
3	Kasper, Lloyd H.	17	University of Michigan (USA)	618
4	Wong, F. Susan	16	Cardiff University School of Medicine (UK)	417
5	Gasbarrini, Antonio	16	Università Cattolica del Sacro Cuore (Italy)	55
6	Luo, Xin M.	15	Virginia Tech (USA)	326
7	Fasano, Alessio	14	Harvard Medical School (USA)	169
8	Triplett, Eric W.	13	University of Florida (USA)	335
9	Shoenfeld, Yehuda	13	Reichman University (Israel)	148
10	Taneja, Veena	12	Mayo Clinic (USA)	231

**Figure 5:**
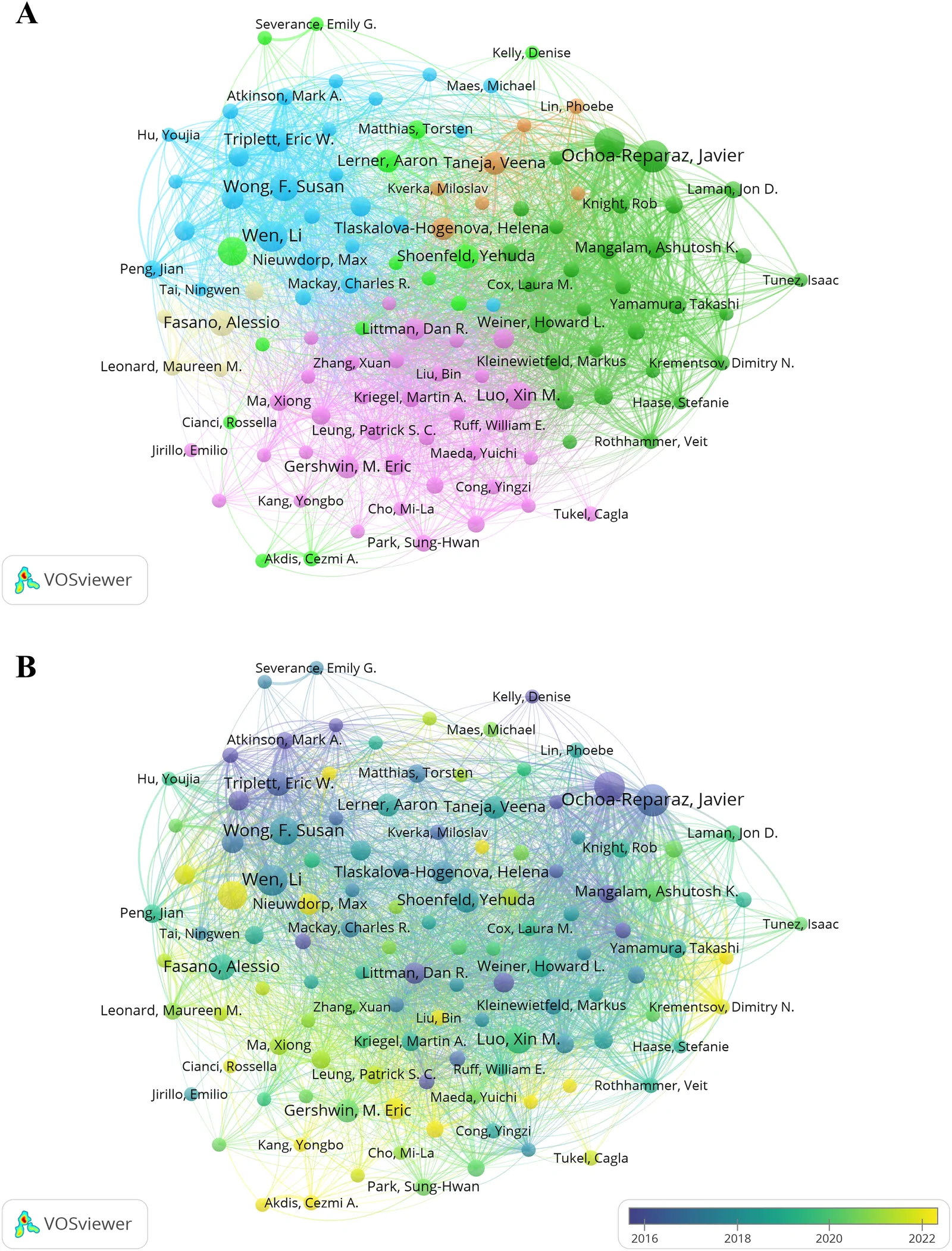
Analysis of gut microbiota and autoimmune diseases-related author. **(A)** Analysis of collaborative network visualization of authors in VOSviewer. **(B)** Analysis of the number of articles published by institutions in recent years.

### Analysis of journals’ distribution and citation collaborative networks

Impact factor (IF) and Journal Citation Reports (JCR) quartiles were used to characterize the academic profiles of the analyzed journals. The top-ranked journals were analyzed based on publication output and co-citation frequency. Frontiers in Immunology (223 articles, IF = 5.7, Q1) published the highest number of articles, followed by International Journal of Molecular Sciences (99 articles, IF = 4.9, Q1) and Nutrients (76 articles, IF = 4.8, Q1). Most highly productive journals were classified as JCR Q1 (**Table 4**).

**Table 4:** Top 10 journals in terms of number of publications, corresponding IF (JCR 2021) and JCR quartile.

Rank	Journals	Publications	IF	JCR quartile
1	Frontiers in Immunology	223	5.7	Q1
2	International Journal of Molecular Sciences	99	4.9	Q1
3	Nutrients	76	4.8	Q1
4	Frontiers in Microbiology	65	4.0	Q2
5	Journal of Autoimmunity	44	7.9	Q1
6	Gut Microbes	42	12.2	Q1
7	Scientific Reports	41	3.8	Q1
8	Frontiers in Cellular and Infection Microbiology	40	4.6	Q2
9	Plos One	39	2.9	Q1
10	Microorganisms	39	4.1	Q2

Co-citation analysis identified Nature (8,608 citations, IF = 50.5, Q1) as the most frequently co-cited journal, followed by Journal of Immunology (6,200 citations, IF = 3.6, Q2) and Proceedings of the National Academy of Sciences of the United States of America (5,841 citations, IF = 9.4, Q1). Most highly co-cited journals had impact factors >5 and were predominantly classified as Q1 journals (**Table 4**; **Supplementary Table 4**).

VOSviewer was used to visualize journal networks related to gut microbiota and autoimmune diseases (**Figure 6**). Journals were classified into eight clusters based on similarity, representing major research areas including immunology, microbiology, nutrition, clinical medicine, molecular biology, and metabolic diseases (**Figure 6A**). Journal co-citation analysis identified six major thematic clusters related to nutrition and microbiology, basic immunology, multidisciplinary biomedical research, gastrointestinal and clinical research, autoimmune and rheumatological diseases, and neuroimmunology (**Figure 6B**). Dual-map overlay analysis demonstrated citation flows mainly from journals in molecular biology, immunology, and clinical medicine to journals in molecular biology and health-related disciplines (**Figure 6C**).

**Figure 6:**
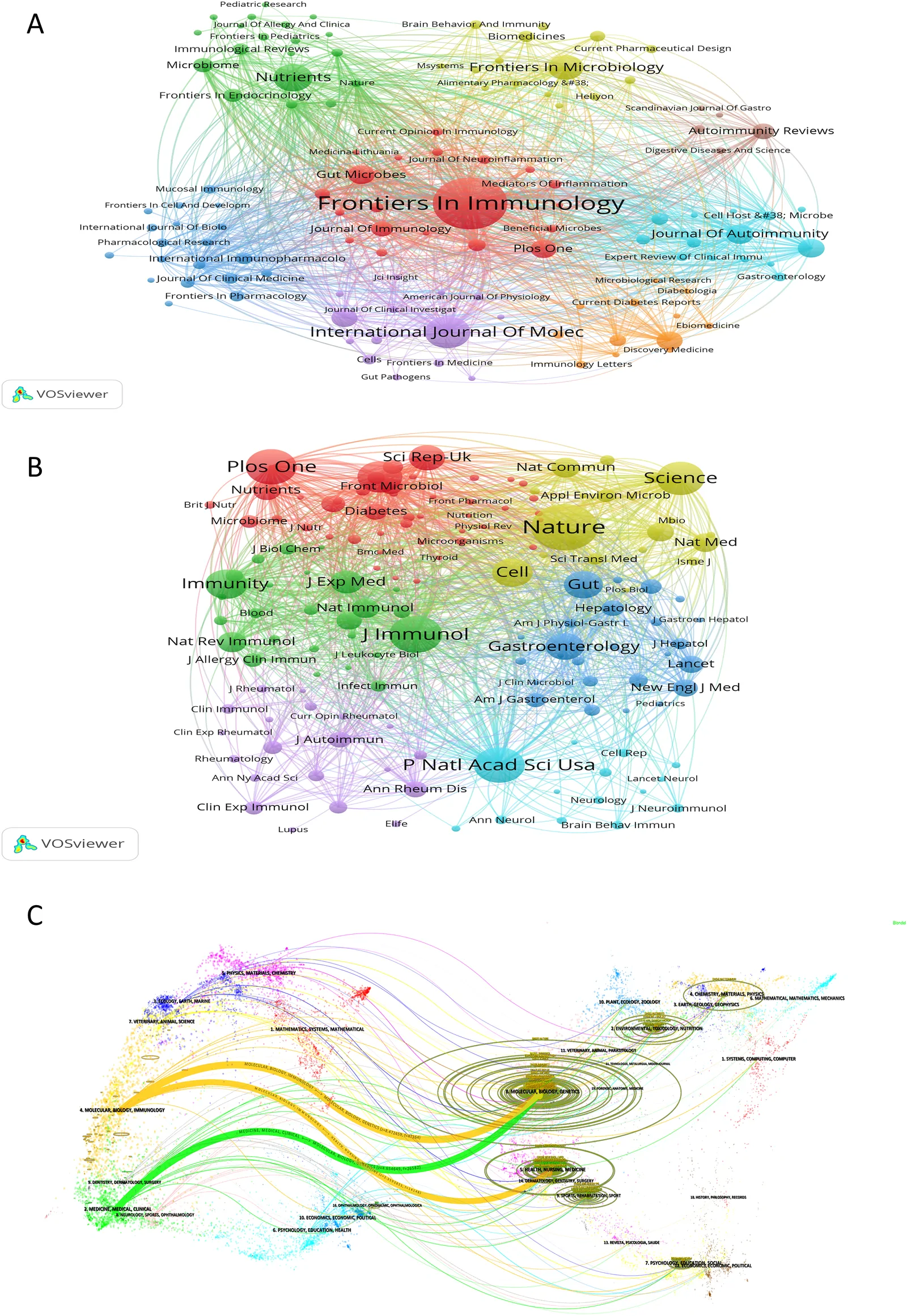
Analysis of gut microbiota and autoimmune diseases-related journal. **(A)** Analysis of collaborative network visualization of journals in VOSviewer. **(B)** Analysis of collaborative network visualization of journals’ citations in VOSviewer. **(C)** The dual-map overlay of journals. Citing journals are on the left, cited journals are on the right, and colored paths indicate citation relationships.

### Keywords analysis

Keyword co-occurrence analysis identified “gut microbiota” (1184) as the most frequently occurring keyword, followed by “microbiota” (516) and “inflammation” (402) (**Table 5**). A keyword co-occurrence network was constructed using VOSviewer to identify major research themes in the field of gut microbiota and autoimmune diseases (**Figure 7A**). In the network, keywords were classified into five thematic clusters representing microbial metabolism, autoimmune diseases, inflammatory bowel disease, rheumatoid arthritis, and probiotic-related research (**Figure 7A**).

**Table 5:** Top 20 keywords in terms of frequency of occurrence and the corresponding total link strength.

Rank	Keyword	Occurrences	Total link strength
1	gut microbiota	1184	5953
2	microbiota	516	2882
3	inflammation	402	2262
4	intestinal microbiota	394	2303
5	microbiome	388	2161
6	autoimmunity	341	2016
7	t-cells	322	1859
8	Regulatory t-cells	305	1827
9	dysbiosis	284	1745
10	disease	283	1449
11	gut microbiome	272	1353
12	chain fatty acids	237	1477
13	probiotics	232	1487
14	inflammatory-bowel-disease	231	1230
15	multiple sclerosis	216	1430
16	bacteria	214	1232
17	Experimental autoimmune encephalomyelitis	210	1214
18	pathogenesis	200	1114
19	expression	198	1007
20	rheumatoid-arthritis	176	975

**Figure 7:**
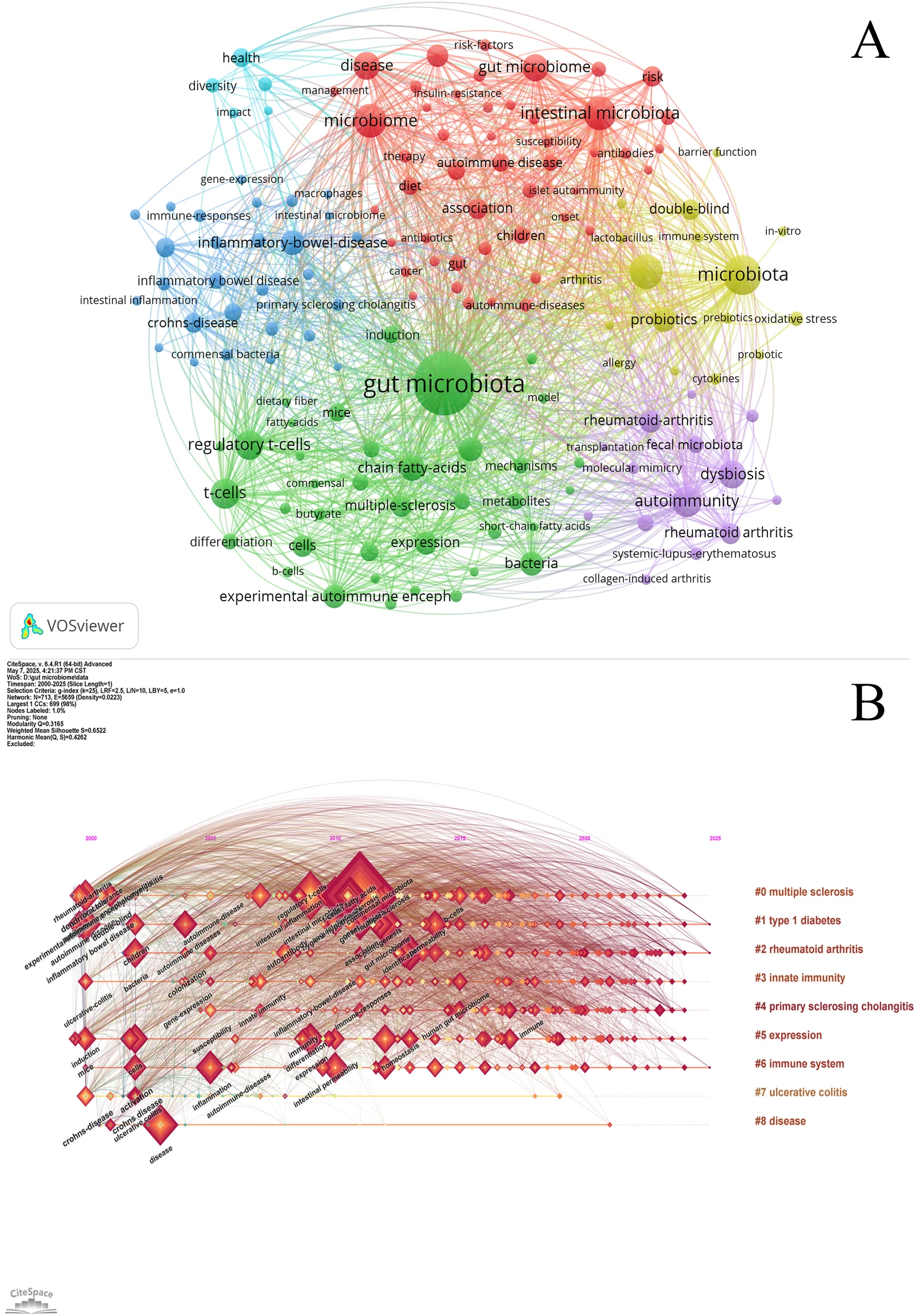
Analysis of gut microbiota and autoimmune diseases-related keyword. **(A)** Collaborative network visualization of keywords in VOSviewer. **(B)** Timeline view of keywords. Each horizontal line represents a cluster. The smaller the number is, the larger the cluster, with #0 being the largest cluster. Nodes size reflects co-citation frequency, and the links between nodes indicate co-citation relationships. Nodes occurrence year is the time when they were first co-cited.

CiteSpace timeline analysis was performed to visualize the evolution of high-frequency keywords across different clusters. As shown in **Figure 7B**, cluster #0 (multiple sclerosis) was the earliest identified and largest cluster. The earliest identified keyword within this cluster was “rheumatoid arthritis”, whereas “gut microbiota” showed the highest weight. Three of the seven clusters remained active research areas, including cluster #1 (type 1 diabetes), cluster #2 (rheumatoid arthritis), and cluster #6 (immune system).

**Figure 8** illustrates the annual popularity trends of keywords from 2000 to 2025. The analysis demonstrated that early research themes, such as “hygiene hypothesis” and “Toll-like receptors”, declined over time, whereas microbiota-related topics remained dominant throughout recent years.

**Figure 8:**
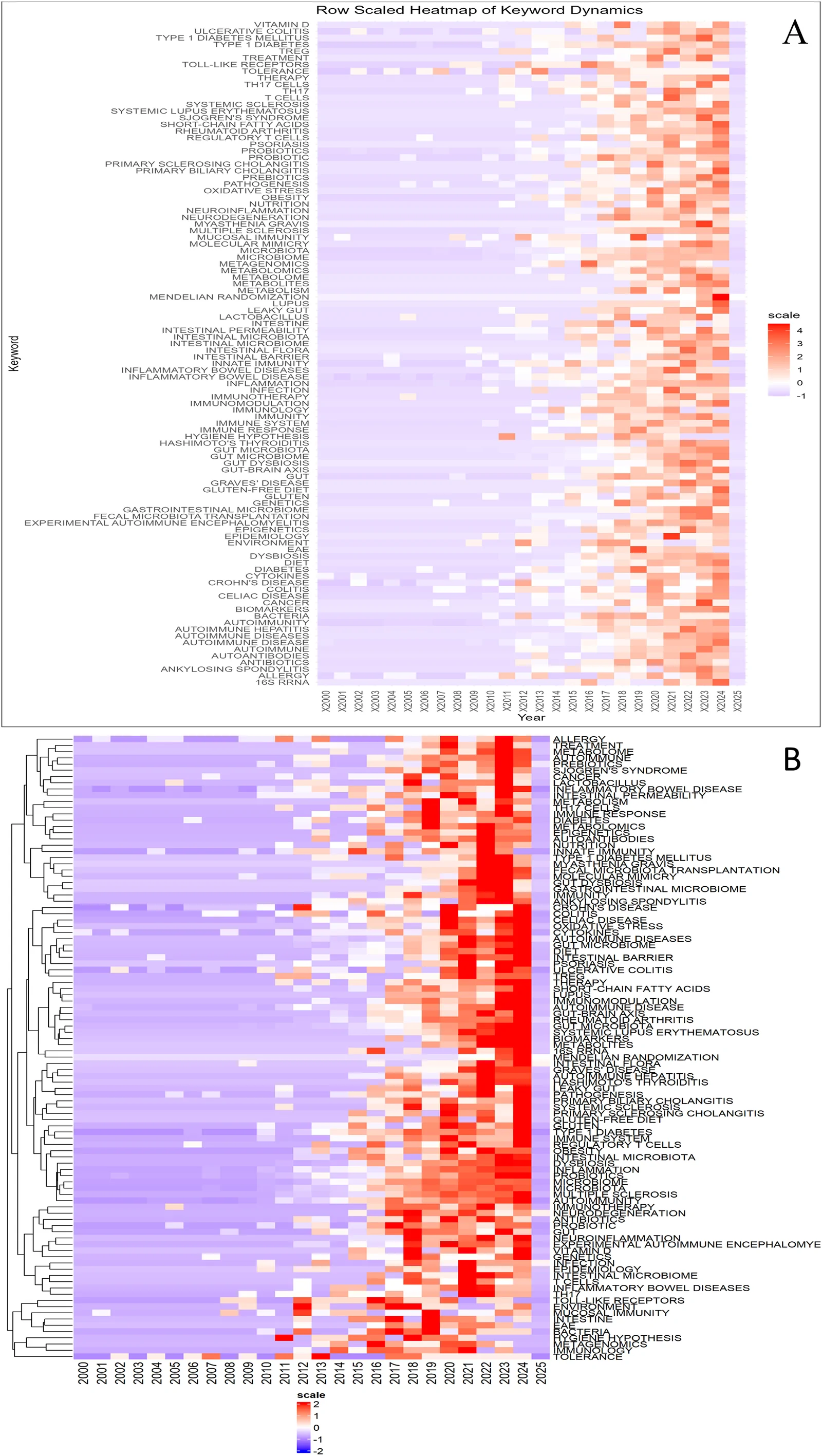
Heatmap analysis of gut microbiota and autoimmune diseases-related keywords. **(A)** Annual heatmap from 2000 to 2025. **(B)** The popularity correlation of keywords.

### Analysis of highly cited literature

**Table 6** summarizes the ten most frequently cited publications in this field. The most cited publication was Ivanov et al. (2009), which reported that segmented filamentous bacteria induce Th17 cell differentiation and promote mucosal immunity (16). The second most cited publication was Belkaid et al. (2014), which focused on the role of microbiota in immune regulation and inflammation (17).

**Table 6:** Top 10 highly cited references.

Rank	Article title	Source title	Author	Year	Cited	DOI
1	Induction of Intestinal Th17 Cells by Segmented Filamentous Bacteria	Cell	Ivaylo I. Ivanov	2009	3413	10.1016/j.cell.2009.09.033
2	Role of the Microbiota in Immunity and Inflammation	Cell	Yasmine Belkaid	2014	3275	10.1016/j.cell.2014.03.011
3	TGF-β-induced Foxp3 inhibits TH17 cell differentiation by antagonizing RORγt function	Nature	Liang Zhou	2008	1544	10.1038/nature06878
4	Innate immunity and intestinal microbiota in the development of Type 1 diabetes	Nature	Li Wen	2008	1509	10.1038/nature07336
5	Sex Differences in the Gut Microbiome Drive Hormone-Dependent Regulation of Autoimmunity	Science	JANET G. M. MARKLE	2013	1440	10.1126/science.1233521
6	Expansion of intestinal Prevotella copri correlates with enhanced susceptibility to arthritis	eLife	Jose U Scher	2013	1410	10.7554/eLife.01202
7	Intestinal barrier function: Molecular regulation and disease pathogenesis	Journal of Allergy and Clinical Immunology	Katherine R. Groschwitz BS	2009	1262	10.1016/j.jaci.2009.05.038
8	The Microbiome in Inflammatory Bowel Disease: Current Status and the Future Ahead	Gastroenterology	Aleksandar D. Kostic	2014	1255	10.1053/j.gastro.2014.02.009
9	Gut-Residing Segmented Filamentous Bacteria Drive Autoimmune Arthritis via T Helper 17 Cells	Immunity	Hsin-Jung Wu	2010	1198	10.1016/j.immuni.2010.06.001
10	The Immunology of Mucosal Models of Inflammation	ANNUAL REVIEW OF IMMUNOLOGY	Warren Strober	2002	1108	10.1146/annurev.immunol.20.100301.064816

The article co-citation analysis was conducted to characterize the knowledge structure and major research directions in the field of gut microbiota and autoimmune diseases (18). Co-citation analysis identified 19 clusters, among which multiple sclerosis, rheumatoid arthritis, and type 1 diabetes were the predominant research areas. Additional clusters were associated with immunoregulatory mechanisms, microbiota-based interventions, and emerging links between gut microbiota and metabolic or respiratory diseases (**Figure 9A**). The clustering analysis demonstrated good structural validity, with a modularity Q value of 0.83 and a weighted mean silhouette score of 0.92, indicating reliable clustering quality and satisfactory internal consistency.

**Figure 9:**
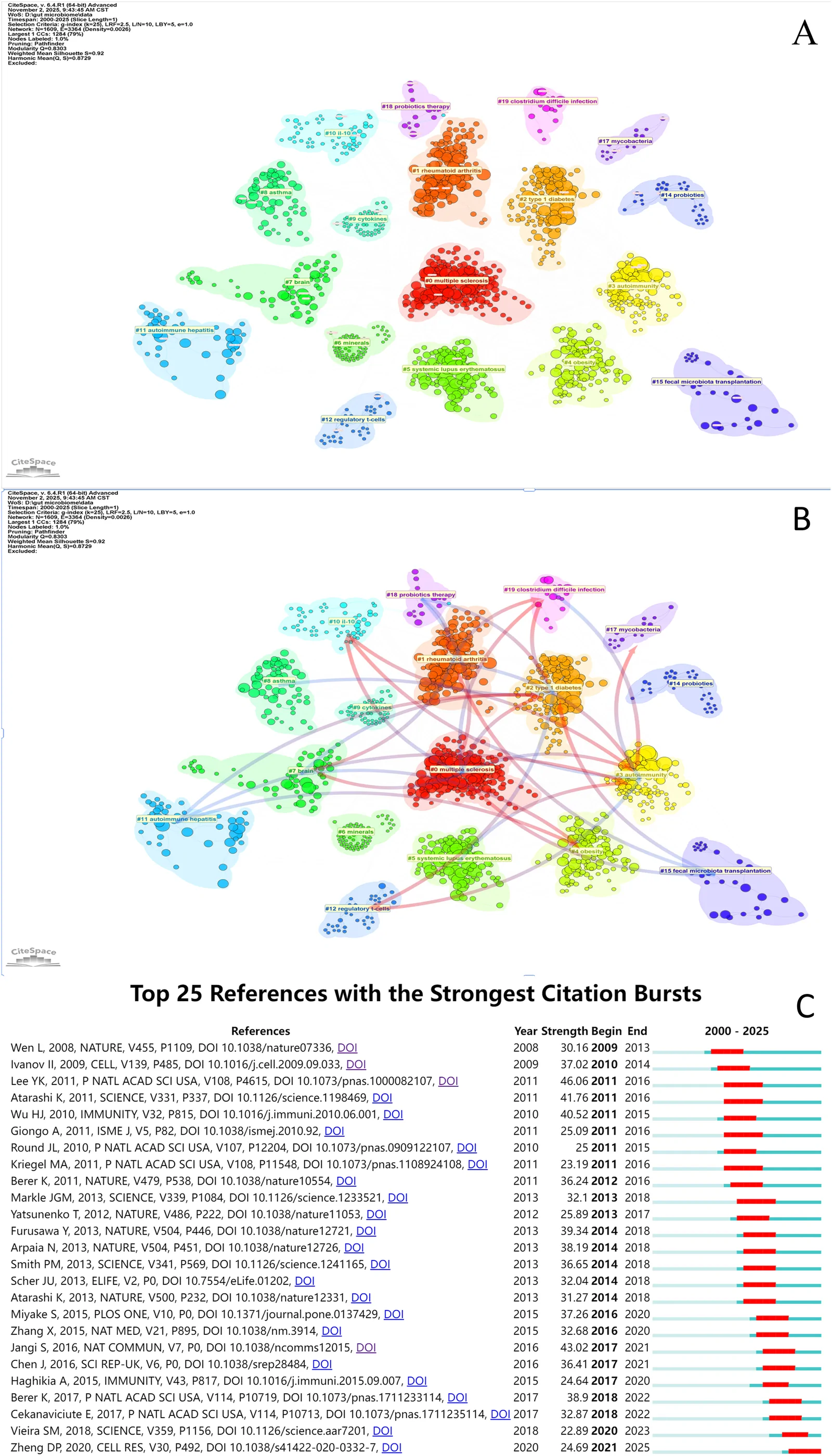
Analysis of gut microbiota and autoimmune diseases-related reference. **(A)** Clustering of references based on similarity between references. The clustering analysis demonstrated good structural validity, with a modularity Q value of 0.83 and a weighted mean silhouette score of 0.92, indicating reliable clustering quality and satisfactory internal consistency. **(B)** Clustering of references based on similarity between references with red and blue arrows. The clusters indicated by red arrows represent the fundamental clusters, while those indicated by blue lines are derived from the clusters indicated by red arrows. The clustering analysis demonstrated good structural validity, with a modularity Q value of 0.83 and a weighted mean silhouette score of 0.92, indicating reliable clustering quality and satisfactory internal consistency. **(C)** Top 25 references with the strongest citation bursts.

**Figure 9B** illustrates the evolutionary relationships among clusters identified by CiteSpace. Clusters #0 (multiple sclerosis), #1 (rheumatoid arthritis), #2 (type 1 diabetes), and #3 (autoimmunity) showed extensive connections with other clusters, indicating their central position in the knowledge evolution network. Red arrows indicated clusters from which subsequent research areas developed, including cluster #10 (“IL-10”), #12 (“regulatory T cells”), and #19 (“Clostridium difficile infection”). In contrast, blue arrows indicated clusters that emerged from previously established research fields, including cluster #11 (“autoimmune hepatitis”), #15 (“fecal microbiota transplantation”), and #18 (“probiotic therapy”).

**Figure 9C** presents the 25 references with the most prominent citation bursts. Burst detection analysis revealed that the strongest citation bursts were mainly observed around 2011 and 2014, corresponding to periods of increased research attention in this field.

### Subject area analysis

CiteSpace analysis revealed citation networks among studies across different subject areas investigating the relationship between gut microbiota and autoimmune diseases (**Supplementary Figure 2**). The literature related to the discipline “IMMUNOLOGY” has the highest number of citations (793), followed by “MICROBIOLOGY” (367). Second after that is “BIOCHEMISTRY &amp; MOLECULAR BIOLOGY”, “GASTROENTEROLOGY &amp; HEPATOLOGY”, “PHARMACOLOGY &amp; PHARMACY”. The influence of disciplines in this field is indicated by the purple circles in **Supplementary Figure 3**. These disciplines also demonstrated high centrality within the co-citation network, indicating their core role in this research field.

### Analysis of clinical experiments in the PubMed database

A PubMed search covering 2000–2024 identified 85 English-language clinical trial reports that met our inclusion criteria. PubMed independently verified the trends presented by WOSCC and also highlighted the significant importance of research in the field of gut microbiota and autoimmune diseases for guiding clinical treatment strategies. **Figure 10A** shows the temporal distribution of publications by country across five consecutive periods from 2000 to 2025. Research output is unevenly distributed across countries, with a small number of contributors accounting for the majority of publications. We can observe that the United States has consistently prioritized the clinical application of basic research findings. United States maintains the highest publication volume throughout the study period, with sustained contributions spanning all time intervals and a marked increase after 2011. China exhibits a pronounced growth trajectory, with most publications concentrated in the two most recent periods, indicating that China has also recognized the importance of clinical application in recent years. Several European countries, including Italy and Germany, contribute consistently across multiple periods, whereas others display more temporally restricted publication patterns. Contributions from countries such as Iran, Japan, India, and Poland remain limited in scale and are largely confined to specific time windows. Overall, the temporal stratification highlights a shift toward increased research output in the later periods, reflecting a broad intensification of research efforts in this field over the past decade. **Figure 10B** shows the distribution of publications across disease categories. Research output is highly uneven, with diabetes, multiple sclerosis, and rheumatoid arthritis accounting for the largest share of publications. Several autoimmune diseases, including celiac disease and systemic lupus erythematosus, exhibit moderate publication frequencies, whereas most other conditions are represented by only a small number of studies.

**Figure 10:**
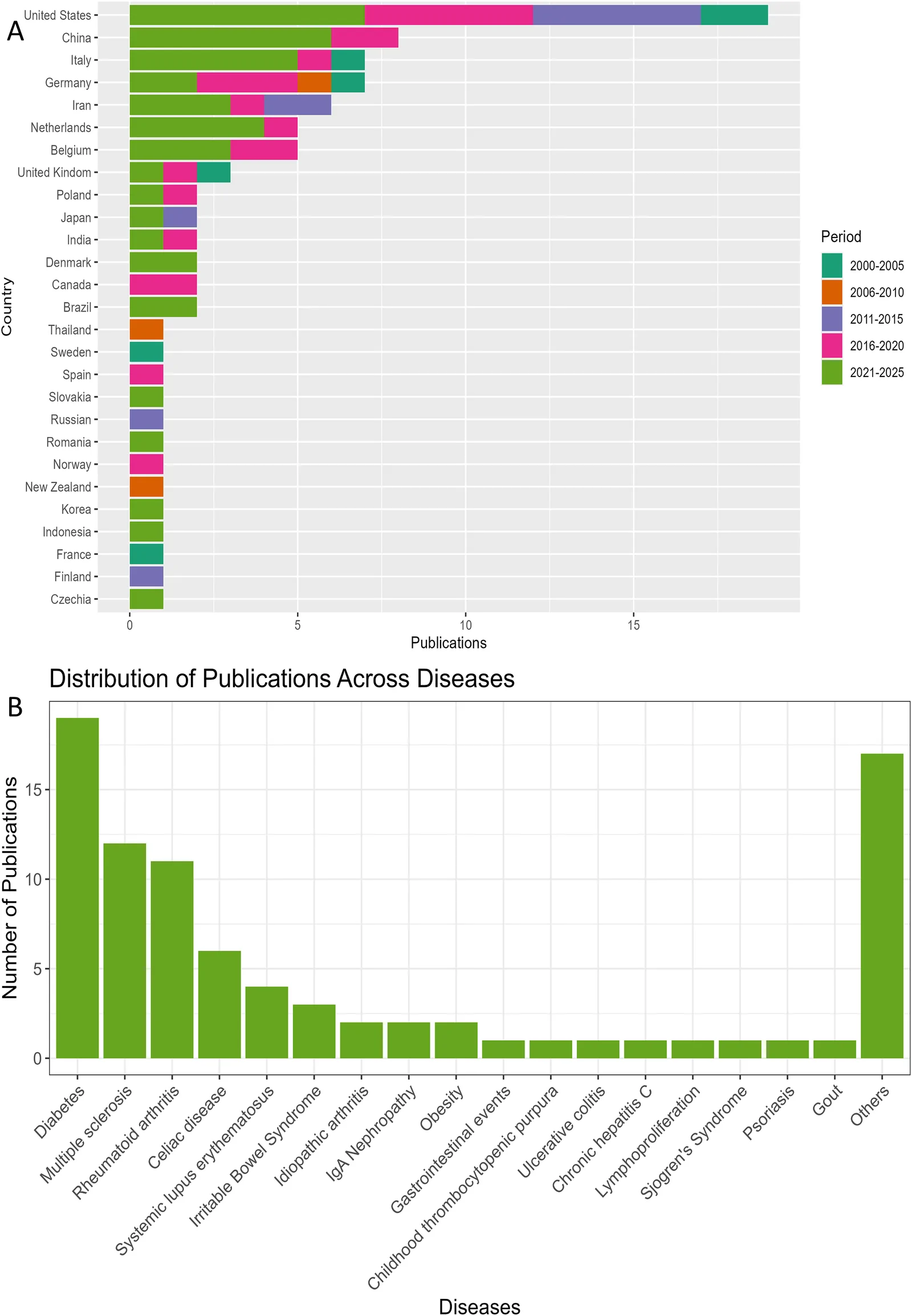
Analysis of gut microbiota and autoimmune diseases-related clinical trials from Pubmed. **(A)** The temporal distribution of publications by country across five consecutive periods from 2000 to 2025. **(B)** The distribution of publications across disease categories.

### Translational landscape from basic microbiome research to clinical applications

The Sankey diagram demonstrated the relationships between major bibliometric hotspots, disease categories, and microbiota-targeted interventions. Gut microbiota dysbiosis showed the strongest connection with clinical studies involving type 1 diabetes mellitus, multiple sclerosis, and rheumatoid arthritis. Probiotic supplementation was the most frequently investigated intervention, followed by dietary interventions, prebiotic/synbiotic approaches, and fecal microbiota transplantation. These findings indicate that current clinical studies have primarily focused on translating microbiome-associated mechanisms into disease-specific therapeutic strategies (**Figure 11**).

**Figure 11:**
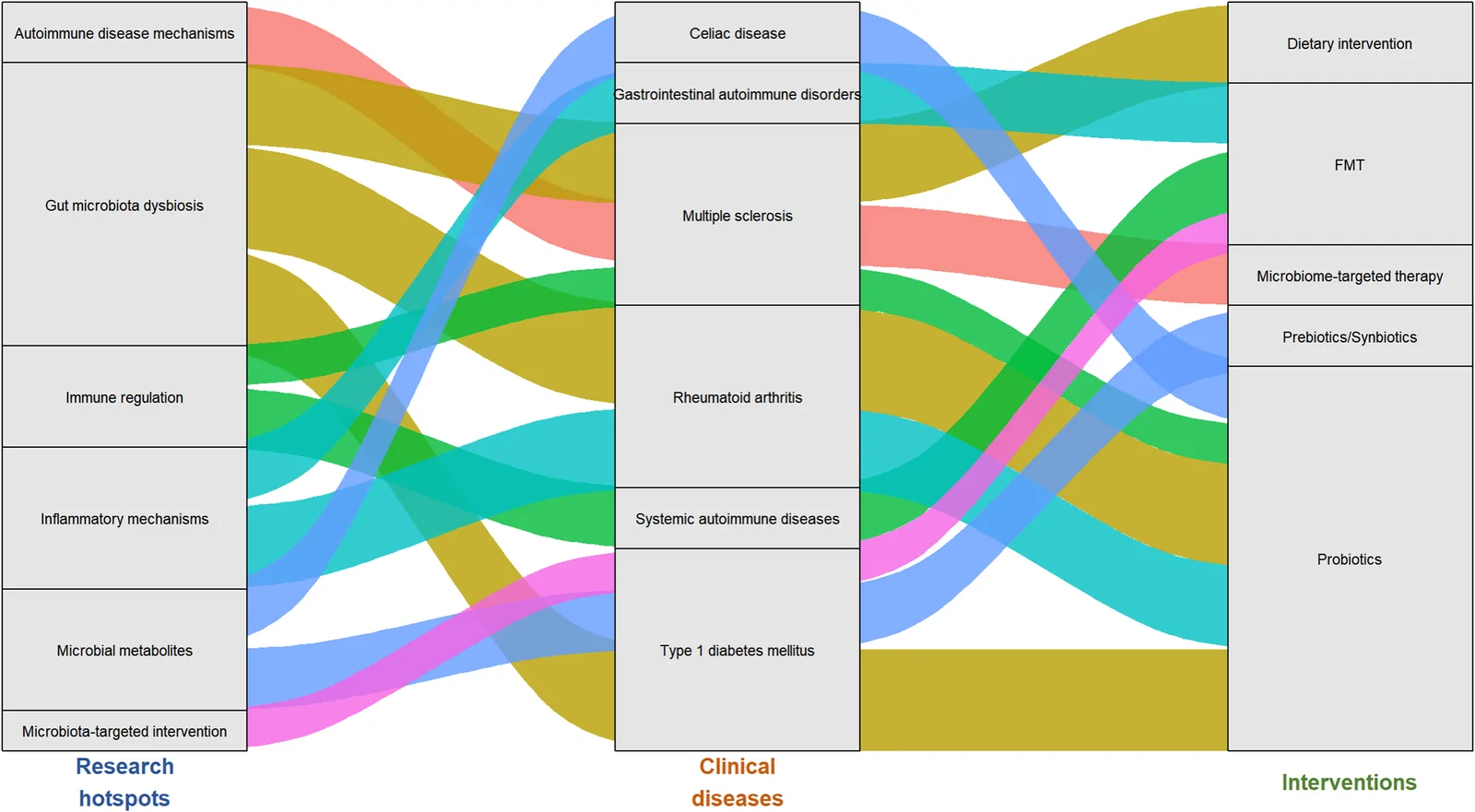
Transition from basic research hotspots to clinical applications in gut microbiota and autoimmune diseases. The Sankey diagram illustrates the translational trajectory from major research hotspots identified through Web of Science bibliometric analysis to autoimmune diseases investigated in PubMed clinical studies and corresponding microbiota-targeted interventions. The width of each flow represents the relative number of studies linking different research themes, disease categories, and therapeutic strategies. This integrated analysis highlights the transition of gut microbiota research from mechanistic exploration toward disease-specific clinical applications.

## Discussion

Although previous bibliometric studies have investigated gut microbiota-related research (19, 20), limited attention has been given to integrating bibliometric findings with clinical evidence in autoimmune disease research. This study extends previous analyses by combining large-scale bibliometric mapping with an assessment of clinical trial evidence to explore the current state of translational research. Key publication characteristics and research themes were analyzed using VOSviewer, CiteSpace, and R (version 4.4.0). The integrated framework provided insights into the evolution of research priorities, collaborative patterns, and translational challenges in this field. Clinical trial data from PubMed were additionally analyzed to evaluate the extent to which basic microbiota research has progressed toward clinical application.

### Global research trends and development trajectory

The continuous increase in publication output and citations indicates sustained scientific interest and rapid development of this research field. The logistic regression model indicated accelerated growth in research output after the mid-2010s, which may be attributed to advances in high-throughput sequencing and systems biology that enabled deeper characterization of gut microbial communities and host–microbe interactions (21, 22). The increased citation activity between 2020 and 2022 suggests growing recognition of microbiota–immune interactions, particularly their relevance to immune dysregulation and autoimmune disease pathogenesis. Collectively, these findings suggest a transition from descriptive characterization of gut microbiota to mechanistic exploration and clinical translation.

### Geographical and institutional collaboration patterns

Publication output and centrality provide complementary perspectives for evaluating research productivity and collaborative influence. In bibliometric networks, centrality reflects the extent to which a country or institution acts as a bridge for knowledge exchange and international collaboration. Research activity in gut microbiota and autoimmune diseases was predominantly concentrated in countries with established biomedical research infrastructure, particularly the United States, China, and several European countries.

The United States represents a leading contributor in publication output, citation impact, and collaboration networks, suggesting strong research capacity and extensive international partnerships. This advantage may be attributed to substantial investment in biomedical research, advanced technological platforms, and well-established interdisciplinary collaborations. China has shown substantial growth in research output in recent years; however, its relatively lower centrality and international collaboration ratio indicate opportunities for further expansion of global partnerships. This pattern is consistent with previous observations in microbiome-related research, suggesting that increasing international cooperation may further enhance the global influence of rapidly developing research communities.

At the institutional level, several leading universities and research centers demonstrated substantial contributions to this field. Harvard University exhibited strong productivity and network connectivity, reflecting its important role in collaborative research and mechanistic investigations of microbiota–immune interactions. Studies from leading institutions have contributed to understanding how microbial communities influence immune regulation and autoimmune disease development, thereby shaping current research directions (23, 24).

Citation-based analyses further identified institutions such as New York University, Harvard University, and the University of California, San Francisco as highly influential contributors. However, relatively limited inter-institutional citation exchange suggests that collaboration networks remain fragmented. This pattern may reflect the influence of geographical, cultural, and communication factors that commonly shape international research collaboration (25, 26). Meanwhile, the increasing contribution of Chinese institutions, including the Chinese Academy of Sciences and Central South University, indicates a growing research presence in Asia driven by expanding investment in microbiome and immunology research.

### Author and journal influence: intellectual foundations of the field

Author network analysis identified several highly productive researchers, including Ochoa-Reparaz, J., Wen, L., and Kasper, L.H., whose contributions have helped shape major research directions in gut microbiota and autoimmune disease research. Their strong collaboration links suggest extensive cooperation among key research groups within this interdisciplinary field. Citation analysis further highlighted influential researchers, including Atarashi, K., Ochoa-Reparaz, J., and Berer, K., reflecting their substantial contributions to understanding microbiota-mediated immune regulation.

Ochoa-Reparaz, J. has made important contributions to elucidating the relationship between gut microbial communities and autoimmune neurological disorders, particularly through studies investigating microbial regulation of immune tolerance and neuroinflammation (27). The predominance of US-based researchers among highly productive authors reflects the strong contribution of American research institutions to this field, while also emphasizing the importance of continued international collaboration.

Journal analysis indicates that influential publications are distributed across journals representing immunology, microbiology, nutrition, and molecular biology disciplines, highlighting the inherently interdisciplinary nature of gut microbiota research. The dual-map overlay suggests an increasing integration of basic biological discoveries with clinical and translational research, reflecting the transition of this field toward microbiota-based therapeutic strategies and precision medicine.

Highly co-cited journals, including Nature, Journal of Immunology, and Proceedings of the National Academy of Sciences of the United States of America, represent key sources of foundational knowledge in this field. These journals have published influential studies that advanced mechanistic understanding of microbiota–immune interactions, including the regulation of autoimmune susceptibility, immune cell differentiation, and host–microbe communication. The presence of highly cited studies from these journals highlights their important role in establishing the conceptual framework of gut microbiota research in autoimmune diseases.

### Hotspots and emerging frontiers

Temporal analysis of burst keywords revealed a progressive evolution of research priorities in the field of gut microbiota and autoimmune diseases, reflecting a transition from descriptive characterization of microbial alterations toward mechanistic investigation and clinical translation. Early research hotspots were primarily associated with gastrointestinal inflammatory disorders, including Crohn’s disease, ulcerative colitis, and inflammatory bowel disease (28, 29). During this initial stage, investigations mainly focused on identifying disease-associated dysbiosis and establishing associations between alterations in gut microbial composition and inflammatory phenotypes, providing the foundation for subsequent exploration of microbiota-mediated immune regulation (30).

From 2010 to 2017, the emergence of keywords such as ‘toll-like receptors’, ‘regulatory T cells’, and ‘innate immunity’ indicated a shift toward mechanistic studies focusing on host–microbe interactions. During this period, increasing evidence from experimental models demonstrated that gut microbiota could influence autoimmune susceptibility by modulating immune tolerance, inflammatory signaling, and immune cell differentiation (31). For example, studies involving specific commensal bacteria and genetically susceptible animal models demonstrated that microbial alterations could reshape immune responses and affect autoimmune disease development (32). These findings contributed to a conceptual transition from simply identifying dysbiosis patterns toward understanding the functional mechanisms underlying microbiota–immune interactions.

Recent keyword bursts (2021–2025), including gut dysbiosis, Mendelian randomization, and management, suggest a further shift toward causal inference and clinical application. The increasing use of Mendelian randomization and human cohort studies reflects growing efforts to distinguish causal relationships from correlations between gut microbiota alterations and autoimmune diseases (33). Meanwhile, the emergence of management-related keywords indicates increasing interest in microbiota-targeted interventions, including dietary modification, probiotics, and personalized microbial therapies (32, 34). However, compared with the substantial mechanistic evidence generated from experimental studies, clinical validation remains limited. The analysis of clinical trials in PubMed further supports the existence of a translational gap between biological discoveries and therapeutic implementation.

The high frequency of keywords related to microbial metabolites, particularly short-chain fatty acids (SCFAs), highlights the increasing recognition of functional microbial products in autoimmune research. Unlike early studies that primarily focused on microbial composition, recent investigations have increasingly emphasized how microbial metabolites mediate host immune regulation (35–39). SCFAs, including acetate, propionate, and butyrate, have been shown to influence immune homeostasis through mechanisms involving regulatory T-cell differentiation, inflammatory signaling modulation, and epigenetic regulation (40–43). The prominence of SCFA-related research reflects a broader transition toward functional interpretation of gut microbiota (44). Nevertheless, most evidence remains derived from experimental models, and the clinical significance of microbiota-derived metabolites requires further validation through well-designed clinical studies.

Immune cell regulation represents another persistent research focus identified through keyword analysis. The frequent occurrence of T-cell-related keywords, particularly regulatory T cells and T helper cells, highlights the central role of adaptive immune responses in microbiota-associated autoimmune research. Current evidence suggests that gut microbial signals contribute to maintaining the balance between immune tolerance and inflammatory activation by regulating regulatory and pro-inflammatory T-cell responses (17, 45–48). Studies investigating microbial taxa capable of inducing Th17 responses, such as segmented filamentous bacteria, have provided important mechanistic insights into how specific microbial communities may influence autoimmune susceptibility (16, 49, 50). However, translation of these findings into human disease remains challenging because of interindividual variability in microbial composition, environmental influences, and differences between experimental models and clinical populations.

In contrast to the relatively broad thematic coverage observed in basic research, the clinical trial literature exhibits a more pronounced concentration across both geographical regions and disease categories. At the national level, clinical trial publications are dominated by a limited number of countries, with the United States contributing consistently across all time periods and China showing marked growth in recent years. This pattern likely reflects differences in clinical research infrastructure, regulatory frameworks, and the capacity to conduct large-scale or multicenter trials, rather than differences in scientific interest alone (51). Disease-specific analysis further reveals that clinical trials are preferentially conducted in a small subset of conditions, particularly diabetes, multiple sclerosis, and rheumatoid arthritis. These diseases are characterized by high prevalence, well-defined diagnostic criteria, and established clinical endpoints, which collectively lower the barriers to trial design and participant recruitment (52). In contrast, many other immune-related or autoimmune conditions are represented by only sporadic trials, suggesting limited clinical translation despite ongoing interest at the preclinical level. Consequently, many autoimmune diseases with strong mechanistic associations with gut microbiota remain underrepresented in clinical investigations. Taken together, these findings indicate that the clinical trial landscape remains substantially more selective than the broader research literature. The observed convergence of trials within specific countries and diseases underscores a translational bottleneck, whereby only a fraction of mechanistic discoveries progress to clinical evaluation. Addressing this imbalance may require expanded international collaboration, harmonization of trial protocols, and targeted support for clinical studies in underrepresented diseases to facilitate more equitable translation of research advances into clinical practice.

### Translational landscape from basic microbiome research to clinical applications

The Sankey diagram illustrates the transition from fundamental microbiome-related research themes to disease-specific clinical investigations and microbiota-targeted interventions (**Figure 10**). The predominant research hotspots identified through bibliometric analysis, including gut microbiota dysbiosis, immune regulation, inflammatory mechanisms, and microbial metabolites, demonstrated clear connections with clinical studies targeting autoimmune disorders. Among these translational pathways, gut microbiota dysbiosis represented the central research theme linking basic microbiome alterations with clinical applications. This hotspot was predominantly associated with type 1 diabetes mellitus, multiple sclerosis, and rheumatoid arthritis, suggesting that microbial imbalance has been widely investigated as a potential contributor to autoimmune pathogenesis across different disease entities (53–55). Regarding therapeutic strategies, probiotics represented the most frequently investigated microbiota-targeted intervention, particularly in patients with type 1 diabetes mellitus and rheumatoid arthritis. Several clinical trials evaluated probiotic supplementation for modulating inflammatory responses, improving metabolic parameters, or influencing disease activity (56, 57). In addition, dietary interventions and prebiotic/synbiotic approaches were increasingly explored, especially in multiple sclerosis and metabolic autoimmune disorders, reflecting growing interest in non-pharmacological microbiome modulation (57). Fecal microbiota transplantation (FMT), although investigated in a smaller number of studies, represented an emerging therapeutic strategy with potential applications in systemic autoimmune diseases and gastrointestinal autoimmune disorders (58). These findings indicate a gradual transition in this field from descriptive characterization of microbial alterations toward microbiota-based therapeutic manipulation. Collectively, the integration of bibliometric findings and clinical trial evidence demonstrates a progressive translational trajectory: early investigations primarily focused on identifying microbial dysbiosis and immune-related mechanisms, whereas recent studies have increasingly shifted toward intervention-oriented approaches aimed at restoring microbial homeostasis and improving autoimmune disease outcomes.

### Multidisciplinary integration and future directions

Subject area analysis indicates that immunology, microbiology, and biochemistry and molecular biology represent the major knowledge foundations of this field, serving as interconnected disciplines that facilitate mechanistic understanding of microbiota–immune interactions. The central position of these disciplines within collaboration networks reflects the inherently interdisciplinary nature of gut microbiota research, which requires integration of microbial ecology, immunological mechanisms, and molecular biology approaches.

The increasing contribution of pharmacology, gastroenterology, and endocrinology suggests that research on gut microbiota and autoimmune diseases is progressively moving toward clinical application and therapeutic development. This multidisciplinary expansion reflects growing recognition that microbial alterations influence not only local intestinal immunity but also systemic immune regulation, metabolism, and disease susceptibility.

Furthermore, the emergence of metabolic and neurological disease-related research themes indicates that gut microbiota studies are expanding beyond traditional autoimmune frameworks toward a broader understanding of systemic immune–metabolic and neuroimmune interactions. Future investigations will likely benefit from integrating multi-omics approaches, including metagenomics, metabolomics, and host immune profiling, to better characterize complex host–microbiota interactions. In addition, stronger collaboration between basic scientists, clinicians, and computational researchers will be essential to facilitate the translation of microbiota-related discoveries into personalized and clinically applicable interventions.

## Limitation

Despite the comprehensive scope of the present bibliometric analysis, several limitations should be acknowledged. First, the analysis was primarily based on publications retrieved from the Web of Science Core Collection and Pubmed, which, although widely recognized for its rigorous indexing standards, may not fully capture all relevant studies in this rapidly evolving field. Second, only English-language publications were included in the analysis. This restriction may have resulted in the exclusion of high-quality studies published in other languages, particularly from regions where research on gut microbiota and autoimmune diseases is actively conducted but less frequently disseminated in English-language journals. As a result, the geographical and thematic diversity of the research output may be partially underestimated. Third, citation-based indicators such as citation counts and centrality measures do not necessarily reflect the true scientific quality or clinical impact of publications, as they can be influenced by publication age, journal visibility, and self-citation. Fourth, keyword-based analyses are subject to inconsistencies in indexing practices and terminology evolution over time, which may affect co-occurrence and clustering results. Forth, the clinical trial analysis was based on available published clinical studies and therefore may not fully represent all ongoing or unpublished clinical investigations.

## Conclusions

This bibliometric analysis maps the evolution of research on gut microbiota and autoimmune diseases over the past two decades, highlighting major transitions from initial characterization of microbial dysbiosis to mechanistic investigation and emerging clinical translation. The field has developed into a highly interdisciplinary research area involving immunology, microbiology, molecular biology, and clinical sciences, with increasing emphasis on understanding how microbial alterations influence immune regulation and autoimmune pathogenesis.

Keyword evolution revealed that current research priorities have shifted toward causal inference, functional interpretation of microbial metabolites, immune cell regulation, and microbiota-targeted therapeutic strategies. However, despite substantial advances in mechanistic understanding, clinical translation remains limited, highlighting the need for well-designed clinical studies, standardized intervention protocols, and personalized approaches.

Overall, this study provides a comprehensive framework for understanding the intellectual structure, developmental trajectory, and future priorities of gut microbiota and autoimmune disease research. Future investigations integrating multi-omics approaches, longitudinal cohort studies, and clinical validation will be essential to translate microbiota-related discoveries into effective strategies for autoimmune disease prevention and treatment.

Future researchers should prioritize causal validation rather than association-based observations and focus on standardized, reproducible microbiome interventions.

## Data Availability

The original contributions presented in the study are included in the article/**Supplementary Material**. Further inquiries can be directed to the corresponding author.

## References

[1] Song Y Li J Wu Y . Evolving understanding of autoimmune mechanisms and new therapeutic strategies of autoimmune disorders. Signal Transduct Target Ther. (2024) 9:263. doi: 10.1038/s41392-024-01952-8 39362875 PMC11452214

[2] Miller FW . The increasing prevalence of autoimmunity and autoimmune diseases: an urgent call to action for improved understanding, diagnosis, treatment, and prevention. Curr Opin Immunol. (2023) 80:102266. doi: 10.1016/j.coi.2022.102266 36446151 PMC9918670

[3] Piccioni A Cicchinelli S Valletta F De Luca G Longhitano Y Candelli M . Gut microbiota and autoimmune diseases: a charming real world together with probiotics. Curr Med Chem. (2022) 29:3147–59. doi: 10.2174/0929867328666210922161913 34551690

[4] Dominguez-Bello MG Godoy-Vitorino F Knight R Blaser MJ . Role of the microbiome in human development. Gut. (2019) 68:1108–14. doi: 10.1136/gutjnl-2018-317503 30670574 PMC6580755

[5] Wang Y Wei J Zhang W Doherty M Zhang Y Xie H . Gut dysbiosis in rheumatic diseases: a systematic review and meta-analysis of 92 observational studies. EBioMedicine. (2022) 80:104055. doi: 10.1016/j.ebiom.2022.104055 35594658 PMC9120231

[6] Guimarães de Oliveira D MaChado A Lacerda PC Karakikla-Mitsakou Z Vasconcelos C . Systemic lupus erythematosus and the gut microbiome: to look forward is to look within - a systematic review and narrative synthesis. Autoimmun Rev. (2025) 24:103921. doi: 10.1016/j.autrev.2025.103921 40876561

[7] Li B Selmi C Tang R Gershwin ME Ma X . The microbiome and autoimmunity: a paradigm from the gut-liver axis. Cell Mol Immunol. (2018) 15:595–609. doi: 10.1038/cmi.2018.7 29706647 PMC6079090

[8] Memariani M Memariani H . Dysbiosis and therapeutic modulation of the gut microbiota in multiple sclerosis: a narrative review. Health Sci Rep. (2025) 8:e71564. doi: 10.1002/hsr2.71564 41341415 PMC12668904

[9] Belvoncikova P Maronek M Gardlik R . Gut dysbiosis and fecal microbiota transplantation in autoimmune diseases. Int J Mol Sci. (2022) 23:10729. doi: 10.3390/ijms231810729 36142642 PMC9503867

[10] Zhu JH Wu LP Deng L Zang SG Li XB Chen X . Gut microbiota and metabolism in systemic lupus erythematosus: from dysbiosis to targeted interventions. Eur J Med Res. (2025) 30:971. doi: 10.1186/s40001-025-03264-1 41088420 PMC12522637

[11] Hicks D Wouters P Waltman L de Rijcke S Rafols I . Bibliometrics: the Leiden Manifesto for research metrics. Nature. (2015) 520:429–31. doi: 10.1038/520429a 25903611

[12] Ninkov A Frank JR Maggio LA . Bibliometrics: methods for studying academic publishing. Perspect Med Educ. (2022) 11:173–6. doi: 10.1007/s40037-021-00695-4 34914027 PMC9240160

[13] Deng P Shi H Pan X Liang H Wang S Wu J . Worldwide research trends on diabetic foot ulcers (2004-2020): suggestions for researchers. J Diabetes Res. (2022) 2022:7991031. doi: 10.1155/2022/7991031 35127951 PMC8813288

[14] Zhang Y Peng Y Xia X . Autoimmune diseases and gut microbiota: a bibliometric and visual analysis from 2004 to 2022. Clin Exp Med. (2023) 23:2813–27. doi: 10.1007/s10238-023-01028-x 36859447 PMC10543628

[15] Xu Y Lyu J Liu H Xue Y . A bibliometric and visualized analysis of the global literature on black soil conservation from 1983–2022 based on CiteSpace and VOSviewer. Agronomy. (2022) 12:2432. doi: 10.3390/agronomy1210243230654563

[16] Ivanov II Atarashi K Manel N Brodie EL Shima T Karaoz U . Induction of intestinal Th17 cells by segmented filamentous bacteria. Cell. (2009) 139:485–98. doi: 10.1016/j.cell.2009.09.033 19836068 PMC2796826

[17] Belkaid Y Hand TW . Role of the microbiota in immunity and inflammation. Cell. (2014) 157:121–41. doi: 10.1016/j.cell.2014.03.011 24679531 PMC4056765

[18] Yang X Wang X Li X Gu D Liang C Li K . Exploring emerging IoT technologies in smart health research: a knowledge graph analysis. BMC Med Inform Decis Mak. (2020) 20:260. doi: 10.1186/s12911-020-01278-9 33032598 PMC7545882

[19] Ni Z Wang S Li Y Zhou L Zhai D Xia D . Mapping trends and hotspot regarding gut microbiota and host immune response: a bibliometric analysis of global research (2011-2021). Front Microbiol. (2022) 13:932197. doi: 10.3389/fmicb.2022.932197 35958122 PMC9361022

[20] Liang J Lin X Liao X Chen X Zhou Y Zhang L . Global bibliometric analysis of traditional Chinese medicine regulating gut microbiota in the treatment of diabetes from 2004 to 2024. Front Pharmacol. (2025) 16:1533984. doi: 10.3389/fphar.2025.1533984 39917613 PMC11799270

[21] Sun X Zhai J . Research status and trends of gut microbiota and intestinal diseases based on bibliometrics. Microorganisms. (2025) 13:673. doi: 10.3390/microorganisms13030673 40142565 PMC11946491

[22] Mo J Su W Qin J Feng J Yu R Li S . Bibliometric and visualized analysis of gut microbiota and hypertension interaction research published from 2001 to 2024. Microorganisms. (2025) 13:1696. doi: 10.3390/microorganisms13071696 40732205 PMC12300951

[23] Kamada N Chen GY Inohara N Núñez G . Control of pathogens and pathobionts by the gut microbiota. Nat Immunol. (2013) 14:685–90. doi: 10.1038/ni.2608 23778796 PMC4083503

[24] Atarashi K Tanoue T Shima T Imaoka A Kuwahara T Momose Y . Induction of colonic regulatory T cells by indigenous Clostridium species. Science. (2011) 331:337–41. doi: 10.1126/science.1198469 21205640 PMC3969237

[25] Wagner CS Park HW Leydesdorff L . The continuing growth of global cooperation networks in research: a conundrum for national governments. PloS One. (2015) 10:e0131816. doi: 10.1371/journal.pone.0131816 26196296 PMC4510583

[26] Moed HF Glänzel W Schmoch U . Handbook of Quantitative Science and Technology Research: The Use of Publication and Patent Statistics in Studies of s&T Systems. Moed HF Glänzel W Schmoch U , editors. Dordrecht: Springer Netherlands (2005). doi: 10.1007/1-4020-2755-9

[27] Ochoa-Repáraz J Mielcarz DW Ditrio LE Burroughs AR Foureau DM Haque-Begum S . Role of gut commensal microflora in the development of experimental autoimmune encephalomyelitis. J Immunol. (2009) 183:6041–50. doi: 10.4049/jimmunol.0900747 19841183

[28] Westall FC . Integrating theories of the etiology of Crohn’s disease on the etiology of Crohn’s disease: questioning the hypotheses. William M. Chamberlin, Saleh A. Naser Med Sci Monit, 2006; 12(2): RA27-33. Med Sci Monit. (2006) 12:LE5–6. 16641883

[29] Brandtzaeg P . Inflammatory bowel disease: clinics and pathology. Do inflammatory bowel disease and periodontal disease have similar immunopathogeneses? Acta Odontol Scand. (2001) 59:235–43. doi: 10.1080/00016350152509265 11570527

[30] Honda K Littman DR . The microbiota in adaptive immune homeostasis and disease. Nature. (2016) 535:75–84. doi: 10.1038/nature18848 27383982

[31] Markle JGM Frank DN Mortin-Toth S Robertson CE Feazel LM Rolle-Kampczyk U . Sex differences in the gut microbiome drive hormone-dependent regulation of autoimmunity. Science. (2013) 339:1084–8. doi: 10.1126/science.1233521 23328391

[32] Xiao YL Gong Y Qi YJ Shao ZM Jiang YZ . Effects of dietary intervention on human diseases: molecular mechanisms and therapeutic potential. Signal Transduct Target Ther. (2024) 9:59. doi: 10.1038/s41392-024-01771-x 38462638 PMC10925609

[33] Sheng D Wang S Li P Li J Xiao Z Lv H . Evidence for genetic causal relationships between gut microbiome, metabolites, and myasthenia gravis: a bidirectional Mendelian randomization study. Front Immunol. (2023) 14:1279845. doi: 10.3389/fimmu.2023.1279845 38179043 PMC10764630

[34] Ginès P Krag A Abraldes JG Solà E Fabrellas N Kamath PS . Liver cirrhosis. Lancet. (2021) 398:1359–76. doi: 10.1016/S0140-6736(21)01374-X 34543610

[35] Yang Z Zhou Y Han Z He K Zhang Y Wu D . The effects of probiotics supplementation on Helicobacter pylori standard treatment: an umbrella review of systematic reviews with meta-analyses. Sci Rep. (2024) 14:10069. doi: 10.1038/s41598-024-59399-4 38697990 PMC11066092

[36] Fitzgibbon G Mills KHG . The microbiota and immune-mediated diseases: opportunities for therapeutic intervention. Eur J Immunol. (2020) 50:326–37. doi: 10.1002/eji.201948322 31991477

[37] Johnson KVA Steenbergen L . Probiotics reduce negative mood over time: the value of daily self-reports in detecting effects. NPJ Ment Health Res. (2025) 4:10. doi: 10.1038/s44184-025-00123-z 40205027 PMC11982403

[38] Salari A Mahdavi-Roshan M Kheirkhah J Ghorbani Z . Probiotics supplementation and cardiometabolic risk factors: a new insight into recent advances, potential mechanisms, and clinical implications. PharmaNutrition. (2021) 16:100261. doi: 10.1016/j.phanu.2021.10026142574925

[39] Abd Elghany K Moustafa MMA Mohamed SA Badr OA Ahmed-Farid OA Hamouda RA . Role of probiotic bacteria against virus pathogenesis: insight of immunological stimulations and possible defence against COVID-19. J Clin Images Med Case Rep. (2024) 5:2866. doi: 10.52768/2766-7820/2866

[40] Kim CH . Complex regulatory effects of gut microbial short-chain fatty acids on immune tolerance and autoimmunity. Cell Mol Immunol. (2023) 20:341–50. doi: 10.1038/s41423-023-00987-1 36854801 PMC10066346

[41] Mann ER Lam YK Uhlig HH . Short-chain fatty acids: linking diet, the microbiome and immunity. Nat Rev Immunol. (2024) 24:577–95. doi: 10.1038/s41577-024-01014-8 38565643

[42] Fung TC Olson CA Hsiao EY . Interactions between the microbiota, immune and nervous systems in health and disease. Nat Neurosci. (2017) 20:145–55. doi: 10.1038/nn.4476 28092661 PMC6960010

[43] Maslowski KM Mackay CR . Diet, gut microbiota and immune responses. Nat Immunol. (2011) 12:5–9. doi: 10.1038/ni0111-5 21169997

[44] Mariño E Richards JL McLeod KH Stanley D Yap YA Knight J . Gut microbial metabolites limit the frequency of autoimmune T cells and protect against type 1 diabetes. Nat Immunol. (2017) 18:552–62. doi: 10.1038/ni.3713 28346408

[45] Sharma A Sharma G Im SH . Gut microbiota in regulatory T cell generation and function: mechanisms and health implications. Gut Microbes. (2025) 17:2516702. doi: 10.1080/19490976.2025.2516702 40517372 PMC12169050

[46] Cortés M Olate P Rodriguez R Diaz R Martínez A Hernández G . Human microbiome as an immunoregulatory axis: mechanisms, dysbiosis, and therapeutic modulation. Microorganisms. (2025) 13:2147. doi: 10.3390/microorganisms13092147 41011478 PMC12472381

[47] Maseda D Manfredo-Vieira S Payne AS . T cell and bacterial microbiota interaction at intestinal and skin epithelial interfaces. Discov Immunol. (2023) 2:kyad024. doi: 10.1093/discim/kyad024 38567051 PMC10917213

[48] Zhou L Lopes JE Chong MMW Ivanov II Min R Victora GD . TGF-beta-induced Foxp3 inhibits T(H)17 cell differentiation by antagonizing RORgammat function. Nature. (2008) 453:236–40. doi: 10.1038/nature06878 18368049 PMC2597437

[49] Flannigan KL Denning TL . Segmented filamentous bacteria-induced immune responses: a balancing act between host protection and autoimmunity. Immunology. (2018) 154:537–46. doi: 10.1111/imm.12950 29771448 PMC6050222

[50] Wu HJ Ivanov II Darce J Hattori K Shima T Umesaki Y . Gut-residing segmented filamentous bacteria drive autoimmune arthritis via T helper 17 cells. Immunity. (2010) 32:815–27. doi: 10.1016/j.immuni.2010.06.001 20620945 PMC2904693

[51] Izarn F Henry J Besle S Ray-Coquard I Blay JY Allignet B . Globalization of clinical trials in oncology: a worldwide quantitative analysis. ESMO Open. (2025) 10:104086. doi: 10.1016/j.esmoop.2024.104086 39700605 PMC11728923

[52] Frazzei G van Vollenhoven RF de Jong BA Siegelaar SE van Schaardenburg D . Preclinical autoimmune disease: a comparison of rheumatoid arthritis, systemic lupus erythematosus, multiple sclerosis and type 1 diabetes. Front Immunol. (2022) 13:899372. doi: 10.3389/fimmu.2022.899372 35844538 PMC9281565

[53] Alkanani AK Hara N Gottlieb PA Ir D Robertson CE Wagner BD . Alterations in intestinal microbiota correlate with susceptibility to type 1 diabetes. Diabetes. (2015) 64:3510–20. doi: 10.2337/db14-1847 26068542 PMC4587635

[54] Jangi S Gandhi R Cox LM Li N von Glehn F Yan R . Alterations of the human gut microbiome in multiple sclerosis. Nat Commun. (2016) 7:12015. doi: 10.1038/ncomms12015 27352007 PMC4931233

[55] Scher JU Sczesnak A Longman RS Segata N Ubeda C Bielski C . Expansion of intestinal Prevotella copri correlates with enhanced susceptibility to arthritis. Elife. (2013) 2:e01202. doi: 10.7554/eLife.01202 24192039 PMC3816614

[56] Maldonado Galdeano C Cazorla SI Lemme Dumit JM Vélez E Perdigón G . Beneficial effects of probiotic consumption on the immune system. Ann Nutr Metab. (2019) 74:115–24. doi: 10.1159/000496426 30673668

[57] Zmora N Suez J Elinav E . You are what you eat: diet, health and the gut microbiota. Nat Rev Gastroenterol Hepatol. (2019) 16:35–56. doi: 10.1038/s41575-018-0061-2 30262901

[58] Paramsothy S Kamm MA Kaakoush NO Walsh AJ van den Bogaerde J Samuel D . Multidonor intensive faecal microbiota transplantation for active ulcerative colitis: a randomised placebo-controlled trial. Lancet. (2017) 389:1218–28. doi: 10.1016/S0140-6736(17)30182-4 28214091

