# Supplementary material for: Mapping research hotspots and trends in gut microbiota and autoimmune diseases since the 21st century: a bibliometric visualization analysis based on two databases
Source: Front Immunol. 2026 Sep 18;17:1793126. doi: 10.3389/fimmu.2026.1793126 (PMC13631007; doi:10.3389/fimmu.2026.1793126)
Supplement: Supplementary file 1 [file Table1.docx]

| **Database** | **Search terms and strategy** |
| --- | --- |
| Web of Science | (TS=(gut) OR TS=(intestine) OR TS=(gastrointestine) OR TS=(gastro-intestine)) AND (TS=(microbiota) OR TS=(microbiome) OR TS=(flora) OR TS=(microflora) OR TS=(bacteria)) AND (TS=(autoimmune disease) OR TS=(autoimmune disorder) OR TS=(autoimmunity) OR TS=(lupus) OR TS=(systemic lupus erythematosus) OR TS=(rheumatoid arthritis) OR TS=(multiple sclerosis) OR TS=(inflammatory bowel disease) OR TS=(Crohn's disease) OR TS=(ulcerative colitis) OR TS=(type 1 diabetes) OR TS=(psoriasis) OR TS=(autoimmune thyroiditis) |
| PubMed | ((gut) OR (intestine) OR (gastrointestine) OR (gastro-intestine)) AND ((microbiota) OR (microbiome) OR (flora) OR (microflora) OR (bacteria)) AND ((autoimmune disease) OR (autoimmune disorder) OR (autoimmunity) OR (Disease, Autoimmune) OR (Diseases, Autoimmune)) |

Supplementary Table 1. The detailed Search terms and strategies for each database.

| **Rank** | **Institutions** | **Citation** | **Centrality** |
| --- | --- | --- | --- |
| 1 | New York University | 11507 | 0.03 |
| 2 | Harvard University | 9656 | 0.11 |
| 3 | University of California San Francisco | 8731 | 0.03 |
| 4 | National Institute of Allergy and Infectious Diseases | 6481 | 0.01 |
| 5 | Yakult Central Institute of Microbiology Research | 6346 | 0.01 |
| 6 | Harvard Medical School | 5470 | 0.08 |
| 7 | University of Tokyo | 5087 | 0.01 |
| 8 | Osaka University | 4548 | 0.01 |
| 9 | Yale University | 3984 | 0.03 |
| 10 | Monash University | 3936 | 0.06 |

Supplementary Table 2 Top 10 institutions in terms of number of frequency of citations and the corresponding centrality.

| **Rank** | **Authors** | **Citations** | **Institutions** | **Total Link Strength** |
| --- | --- | --- | --- | --- |
| 1 | Atarashi, K | 655 | Keio University School of Medicine (Japan) | 19798 |
| 2 | Ochoa-reparaz, J | 630 | Boise State University (USA) | 21243 |
| 3 | Berer, K | 587 | Max Planck Institute of Neurobiology (Germany) | 17820 |
| 4 | Scher, Ju | 563 | New York University (USA) | 13878 |
| 5 | Ivanov, Ii | 554 | Columbia University (USA) | 15125 |
| 6 | Round, Jl | 482 | University of Utah (USA) | 14303 |
| 7 | Chen, J | 463 | Dalian Polytechnic University (China) | 12558 |
| 8 | Turnbaugh, Pj | 424 | University of California (USA) | 11963 |
| 9 | Lee,Yk | 376 | Hallym University College of Medicine (Korea) | 11046 |
| 10 | Wu, Hj | 365 | University of New South Wales (Sydney) | 10004 |

Supplementary Table 3 Top 10 authors in terms of number of citations, the corresponding institutions and total link strength.

| **Rank** | **Journals** | **Co-citations** | **IF** | **JCR quartile** |
| --- | --- | --- | --- | --- |
| 1 | Nature | 8608 | 50.5 | Q1 |
| 2 | Journal of Immunology | 6200 | 3.6 | Q2 |
| 3 | Proceedings of the National Academy of Sciences of the United States of America | 5841 | 9.4 | Q1 |
| 4 | Plos One | 5765 | 2.9 | Q1 |
| 5 | Science | 5343 | 44.8 | Q1 |
| 6 | Frontiers in Immunology | 4873 | 5.7 | Q1 |
| 7 | Immunity | 4431 | 25.5 | Q1 |
| 8 | Cell | 4223 | 45.6 | Q1 |
| 9 | Gut | 3949 | 23.1 | Q1 |
| 10 | Gastroenterology | 3871 | 26.3 | Q1 |

Supplementary Table 4 Top 10 journals in terms of number of co-citations, corresponding IF (JCR 2021) and JCR quartile.
